# Artificial intelligence and machine learning models for predicting and evaluating the influence of shelf-life environments and packaging materials on garlic (*Allium Sativum* L) physicochemical and phytochemical compositions

**DOI:** 10.1016/j.fochx.2025.102731

**Published:** 2025-07-06

**Authors:** Hany S. El-Mesery, Ahmed H. ElMesiry, Mansuur Husein, Zicheng Hu, Ali Salem

**Affiliations:** a School of Energy and Power Engineering, Jiangsu University, Zhenjiang 212013, China; b Agricultural Engineering Research Institute, Agricultural Research Center, Dokki, 12611 Giza, Egypt; c Faculty of Computer Science and Engineering, New Mansoura University, 35742, Egypt; d School of Environment and Safety Engineering, Jiangsu University, Zhenjiang 212013, China; e Department of Water and Environmental Engineering, Faculty of Engineering, Tamale Technical University, P.O. Box 3 E/R, Tamale, Ghana; f Civil Engineering Department, Faculty of Engineering, Minia University, Minia 61111, Egypt; g Structural Diagnostics and Analysis Research Group, Faculty of Engineering and Information Technology, University of Pécs, Pécs 7622, Hungary

**Keywords:** Machine learning, Storage, Phytochemical, Physicochemical, Artificial intelligence, garlic

## Abstract

The nutritional content and quality of garlic, a crop widely consumed, must be preserved after harvesting by overcoming several challenges. The necessity of this study arises from the growing demand for effective postharvest management solutions that can extend shelf life, maintain the nutritional integrity of garlic and enhance consumer satisfaction. This study explores the application of machine learning (ML) and artificial intelligence (AI) to predict the effects of storage environments and packaging materials on garlic's physicochemical properties, enzymatic activities, phytochemical content, and antioxidant characteristics. The results show that temperature significantly influenced most parameters, except for anthocyanin, and that packaging impacted all variables. Storing garlic at 4 °C was found to preserve its quality better than at 25 °C, offering insights into optimizing storage conditions and packaging for superior product quality. This research provides valuable guidance on controlling factors that affect garlic's postharvest performance, aiming to improve preservation and reduce food waste.

## Highlights

• Integrating machine learning models with AI-assisted optimization improves garlic postharvest management.
• The temperature of the storage environment influenced all parameters except anthocyanin.
• Packaging conditions and temperature influenced all proportions except total color changes.
• ML models showed that storing garlic at 4 °C yields better quality than at 25 °C.
• Optimizing storage and packaging is essential to preserve garlic's quality and nutrients.

## 1 Introduction

Garlic is a vital plant utilized worldwide for culinary and therapeutic applications. It is the most cost-effective species within the Allium genus, serving as a key vegetable crop and a frequently used ingredient in cooking. Due to its therapeutic and nutritional properties, garlic has attracted significant interest in both traditional and modern healthcare practices. Despite its widespread use, the post-packaging quality of garlic products, especially throughout the distribution chain, is often assessed through subjective sensory evaluation, which can be inconsistent and unreliable. A primary challenge in preserving garlic cloves is the facilitation of shoot germination and rooting, a process that is heavily influenced by factors like temperature and storage duration. Elevated humidity levels and inadequate plastic packaging can exacerbate these issues, resulting in degraded product quality during storage (Yan et al., 2021).

Garlic has historically been utilized in its raw form, including dried cloves and new leaves, and in various processed forms like dried oil, powder, and extract. The unique flavor profile of cloves can be attributed to compounds containing sulfur and non-volatile amino acids. Furthermore, alliin, also known as S-allyl-cysteine sulfoxide (ACSO), is the primary precursor for garlic flavor (Lu et al., 2022). Upon the smashing or cutting of garlic cloves, the cell membrane disruption occurs, leading to the release of Allinase, an intact enzyme found within the vacuole of garlic cells. Allicin is the main result of this enzyme's subsequent reaction with alliin. In contrast to light, the allicin in the liquid extract showed sensitivity to pH and temperature during storage (El-Mesery, Adelusi, et al., 2024). Conversely, garlic exhibited a significant concentration of vitamins, especially those from the B complex and vitamin C, alongside essential minerals like phosphorus, potassium, and selenium, as well as phenolic compounds, including flavonoids, phenolic acids, and nitrogen-containing substances. Numerous pre-harvest and postharvest factors can affect garlic's chemical and phytochemical compositions (El-Mesery, Qenawy, et al., 2024).

Consequently, the conditions under which storage occurs- such as temperature, duration, humidity, and the materials used for packaging- play a crucial role in influencing postharvest processes. These variables can negatively impact the physicochemical properties, enzyme activities, chemical compound structure, antioxidant capabilities, and nutritional benefits of garlic (Cui et al., 2019). The qualities of garlic, including texture, firmness, and flavor, have a significant influence on consumer acceptability and preference. In specific cases, visually appealing garlic that offers superior texture and flavor is often perceived as a luxury product, thereby enhancing the customer experience and increasing market demand. Moreover, enzymes play a critical role in the ripening and degradation processes that occur after harvesting, significantly altering the characteristics of garlic during storage. Temperatures exceeding −2 °C, which is considered the ideal storage temperature, result in elevated CO_2_ levels that hinder germination and promote root growth in high-moisture conditions (Huang, Ding, et al., 2018).

The activity of enzymes responsible for breaking down garlic cell wall components is influenced by temperature, which impacts their physiochemical properties, particularly firmness and texture (Rashid et al., 2018). Elevated humidity levels can facilitate the proliferation of mold and spoilage microorganisms, thereby hastening the deterioration of garlic. On the other hand, low humidity aids in minimizing excess moisture on the garlic's surface, thereby decreasing the likelihood of rot and extending its shelf life. As individuals grow more aware of their health, there is a rising interest in garlic, which offers substantial nutritional benefits and a longer shelf life. Nonetheless, reaching these objectives presents various challenges, such as determining the ideal storage environments and selecting appropriate packaging products to maintain the garlic's physiochemical (Xiong et al., 2018).

Introducing machine learning algorithms for forecasting and AI-based techniques has created new opportunities across various industries, including agriculture. Algorithms are employed in AI-driven efficiency to determine the best solutions for complex issues, such as selecting optimal packing materials and storage conditions that preserve the quality of agricultural products. Likewise, artificial intelligence algorithms utilize vast databases to identify trends and relationships, enabling accurate forecasts and informed decisions. In recent years, researchers have become increasingly aware of these tools' potential to tackle challenges related to storage environments and packing supplies that impact the overall quality and nutritional content of foods (El-Mesery, Qenawy, et al., 2024). One can create predictive models and use Artificial Intelligence (AI) optimization to find the best conditions for preserving garlic quality by applying machine learning techniques and intelligence-based optimization to large datasets of postharvest garlic environments This tactic will ensure that customers purchase garlic in the best possible circumstances. This work estimates the influences of storage environments, precisely temperature, duration, and packing bags, on garlic's physicochemical attributes, enzyme activities, phytochemicals, and antioxidant features. The research also aims to optimize and predict garlic's physiochemical attributes using artificial intelligence and machine learningC.

## 2 Methodology

### 2.1 Materials

Samples of garlic were collected from a local market in Zhenjiang, Jiangsu Province, China. The garlic bulbs were carefully peeled by hand, ensuring they were of uniform size and free from pests and any damage. The garlic cloves were stored in a laboratory at 4 °C before application. After meticulously examining the garlic, those damaged or rotten were removed. The remaining samples were categorized by shape and size, facilitating efficient packaging and storage.

### 2.2 Shelf-life conditions and packaging materials

500 g of garlic cloves were carefully sorted, cleaned, and then packed using three different materials: paper bags (PB), perforated high-density polyethylene (P-HD), and non-perforated high-density polyethylene (HD). The storage condition was specified with two (2) distinct extreme temperatures, 4 °C and 25 ± 1 °C, which were used to preserve the cloves for 30 days. A control group that did not receive any packaging (WP) was also established for every storage temperature. The garlic's physicochemical characteristics and hardness were evaluated every 0 to 5 days for 30 days. At the beginning and end of the test period (30th day), the cloves' color, enzyme activity, phytochemicals, and antioxidant properties were examined exclusively.

### 2.3 Analysis of physicochemical properties

Garlic juice samples were obtained by blending 10 g of garlic cloves with 50 mL of distilled water using a homogenizer (Ultra-Turrax, IKA, Germany) at 12,000 rpm for 2 min at 4 °C. The homogenate was then filtered through Whatman No. 1 filter paper, and the clear extract was used for physicochemical analyses. The measurements included total soluble solids (TSS), titratable acids (TA), and pH. TSS was measured at 20 °C using a handheld refractometer made by ATAGO (Tokyo, Japan). pH was measured with a calibrated digital pH meter (Mettler Toledo, USA) at 25 °C. Titratable acidity was determined by mixing 10 g of the homogenate with 100 mL of water, followed by titration with 0.1 mol/L of Sodium Hydroxide (NaOH), and expressed as citric acid equivalents, as citric acid is a commonly used reference for acidity in food products. The TA is represented as the percentage of citric acid, and pH was also measured using a potentiometer set to 25 °C (W. Wang et al., 2015).

### 2.4 Analysis of moisture content and weight loss

The garlic's moisture percentage was assessed using the microwave technique (AOAC, 2000). A 20 g piece of garlic was based on a pre-dried aluminum sheet for four hours at a temperature of 105 °C. Several factors, both before and after harvesting, affect fruit weight reduction, including the timing of the harvest and storage temperature. Garlic quality can be indicated by weight loss, as a significant decrease results in the tissue becoming soft and dull. The garlic's moisture content (MC) was determined using Eq. 1 (Salehi et al., 2022).

(1) $$\mathrm{MC}=\frac{W_{i}-W_{d}}{W_{d}}$$

W_d_ and W_i_ denote the garlic's dried and initial weights, respectively.

To measure the garlic's weight loss, a durable electrical weighing balance was utilized to assess the garlic's mass at the start of the experiment and at various time intervals throughout the storage period. The results were reported as a percentage of the initial weight loss, following Eq. 2.

(2) $$\mathrm{Weight Loss}=\frac{\mathrm{initial weight}-\mathrm{Final weight}}{\mathrm{Final weight}}\times 100\%$$

### 2.5 Firmness determination

Firmness is essential for evaluating garlic quality and influencing a buyer's choice when selecting freshly harvested garlic. It serves as a key texture parameter for assessing a material's mechanical resistance when subjected to an applied force. Commonly evaluated in food, pharmaceuticals, cosmetics, and industrial materials, firmness testing helps determine product quality, consistency, and usability (Huang, Pan, et al., 2018). After removing the garlic cloves from the storage compartment, they were processed into uniform samples using a double cutter and punch, measuring 15 mm in diameter and 10 mm in thickness. The Texture Profile Analysis (TPA) mode was employed during testing at room temperature (25 °C) with a texture analyzer (Stable Micro System TA-XTPL, UK). According to (He et al., 2019), the test conditions were as follows: the sample block underwent two compression processes utilizing a type P-25 cylindrical probe. The pretest speed was set at 0.1 cm/s, the test speed at 0.5 cm/s, and the post-test speed at 0.1 cm/s. The strain was 60 %, and the trigger force was 5 g, with the test conducted over 5 replicates.

### 2.6 Color measurement

A movable colorimeter (CR-400 Chroma Meter, Konica Minolta, USA) was utilized to measure the color variation of garlic. Following the calibration with a standard white plate, five assessments were conducted on the four samples across the various storage conditions. Triple measurements were employed to acquire the L, a, and b values. The total variation in color (δE) was calculated using Eq. 3 to analyze the sample's color variation compared to the control.

(3) $$\mathrm{\delta E}=\sqrt{{\left(L^{*}-L_{o}^{*}\right)}^{2}+{\left(a^{*}-a_{o}^{*}\right)}^{2}+{\left(b^{*}-b_{o}^{*}\right)}^{2}}$$

where a, b, and L represent redness/greenness values, yellowness/blueness, and whiteness respectively. The color value of the garlic is denoted by the subscript “0”. When the value of δE is high, it signifies larger change in the color of the blanched sample.

### 2.7 Valuations of enzyme functionality

#### 2.7.1 Ascorbic acid oxidase (AAO), polyphenol oxidase (PPO) and peroxidase (POD) extraction

The enzymatic activity of the fresh samples was evaluated in triplicate for AAO, PPO, POD, and pectin methyl esterase (PME). The extract was obtained following a technique by (W. Wang et al., 2015). A mixture was prepared by combining 5 g of crushed garlic with 10 ml of a buffered phosphate solution at pH 7.0, which contained 1 % (*w*/*v*) polyvinylpolypyrrolidone. The extract was homogenized at 12,000 rpm for 1 min at 4 °C to ensure uniform distribution of components before further enzymatic analysis. The solution underwent a pressing process for 20 min at a temperature of 4 °C, applying a force of 3214 g. The AAO, PPO, and POD activities were evaluated following the methodology outlined by (Jiang et al., 2022). The enzyme activity was measured in units per kilogram (U/kg) of protein, indicating the amount of enzyme required to achieve a 0.001 absorbance per minute for POD, PPO, and AAO. Enzyme activities were measured based on the rate of change in absorbance per minute at specific wavelengths. This formula adheres to the methodology established by (Jiang et al., 2022) and ensures consistent enzyme activity measurements.

The formula for Enzyme activity (U/kg protein) in Eq. 4:

(4) $$\mathrm{Enzyme activity}=\frac{\mathrm{\Delta A}\times V\times 1000\;}{\varepsilon \times d\times W\;}$$

Δ*A* is the change in absorbance per minute; *V* is the total volume of extract (mL); *ε* is the molar extinction coefficient of the enzyme-specific substrate; *d* is path length (cm); and *W*is weight of the sample (g).

#### 2.7.2 Pectin methylesterase (PME) and enzyme assays

Pectin methylesterase (PME) was developed using the methods described by (Grsic-Rausch & Rausch, 2004) with minor modifications. In conclusion, 0.020 L of a 1 % pectin-salt solution was mixed with 0.005 kg of garlic and stored at 30 °C. 2 N NaOH was added to the solution to bring its pH down to 7. The pH was then adjusted to 7.7 using NaOH (0.05 N), and the amount of time needed to get the pH back to 7.0 with 0.0001 L of NaOH (0.05 N) was noted. Using Eq. (5), the pectin methyl esterase enzyme unit (U/mL) was calculated. One unit of enzyme activity is defined as the number of micro equivalents of methyl ester groups cleaved per second under the assay condition.

(5) $$\mathrm{PME}=\frac{\left(0.05N\;N_{a}\mathrm{OH}\right)\;x\;\left(0.5\;\mathrm{mL}\;N_{a}\mathrm{OH}\right)}{\left(0.005\;\mathrm{kg}\;\mathrm{sample}\right)x\left(\mathrm{time}/\min\right)}$$

### 2.8 Determination of antioxidants and phytochemicals

#### 2.8.1 Garlic extract preparation

The garlic specimens were meticulously ground into a fine powder after drying. Twenty milliliters of 96 % ethanol were utilized to extract two grams of garlic. The solution underwent incubation for 12 h at a temperature of 30 °C, with a rotation speed of 150 rpm. The mixtures were subsequently filtered with filter paper, facilitating the application of a ferric-reducing antioxidant power (FRAP) assay to evaluate the quantities of anthocyanins, the total amount of phenolic (TPC), overall flavanol content (TFC), and the activity of antioxidants. Specimens of garlic cloves were meticulously ground into a fine powder.

#### 2.8.2 Evaluation of Total phenolic content

The method described by (Sheng et al., 2019) was employed, with slight adjustments, to assess the total phenolic content (TPC) of the extracted substance using the Folin–Ciocalteu reagent. A combination of two hundred microliters of tomato extract at 0.5 mg/mL concentration with five milliliters of 0.2 mol/L Folin–Ciocalteu reagent. After four minutes, four milliliters of a 7.5 % sodium carbonate solution was added. The absorbances at 760 nm were recorded with an ultraviolet radiation (UV) spectrophotometer (TU-1810; Purkinje General Instrument Co., Ltd., Beijing, China) throughout a 90-min incubation period at 25 °C. The concentration of TPC, measured in milligrams of gallic acid equivalent (GAE) per 100 g of material, was determined using the gallic acid standard curve.

#### 2.8.3 Analysis of total flavanol content

To find the total flavanol content (TFC), 0.2 ml of extract garlic were thoroughly mixed with three milliliters of 0.1 % Dimethylaminocinnamaldehyde (DMACA) solution. The UV spectrophotometer measured the absorbances at 640 nm following a 10-min dark incubation period at 25 °C. (Pu et al., 2013) state that the Rutin standard curve was used to compute the total flavonoid content (TFC) concentration, expressed as mg Rutin equivalent (RE)/100 g sample.

#### 2.8.4 Analysis of anthocyanin

The anthocyanin concentration was assessed using the pH discrepancy technique (Choi et al., 2014). The garlic extract was diluted 20 times, utilizing 0.025 mol/L potassium chloride solution at pH 1.0 and 0.4 mol/L sodium acetate as the buffer at the pH level of 4.5, respectively. The 530 and 700 nm wavelengths' absorbances were subsequently quantified using a UV spectrophotometer. The anthocyanin content was estimated using Eq. 6, presented in milligrams per 100 g of the sample.

(6) $$\mathrm{Total anthocyanin}=\frac{\left(A\times \mathrm{MW}\times \mathrm{DF}\times 1000\right)}{\left(\varepsilon \times 1\right)}$$

In this scenario, MW represents the molecular mass of cyanidin-3-glucoside (449.2 g/mol), DF denotes the dilution factor, ε indicates the molar absorption coefficient value of cyanidin-3-glucoside (26,900 L/mol × cm), and A is calculated as the difference between pH 1.0 (A530 nm − A700 nm) and pH 4.5 (A530 nm − A700 nm).

#### 2.8.5 Determination of ferric-reducing antioxidant power (FRAP)

Just like other natural or plant-based products, they are intrinsically endowed with anti-oxidation potential, which aids in anti-microbial activities. To achieve the purpose of this study, the antioxidant capabilities of garlic extract were assessed using the FRAP test. The ferric-reducing antioxidant power (FRAP) test was carried out using a mixing solution that consisted of 25 mL of 300 mol/L acetate buffer (pH 3.6), 2.5 mL of 10 mol/L 2,4,6-tripyridyl-*S*-triazine (TPTZ) in 40 mol/L HCl, and 20 mL of 20 mol/L FeCl₴·6H₂O. To make sure the reagent was fully activated, the mixture was kept at 37 °C for half an hour. Afterwards, 2.85 mL of the FRAP working solution was mixed with 150 μL of garlic extract (0.2 mg/mL) and left to incubate in the dark at 25 °C for 30 min. Next, an ultraviolet (UV) spectrophotometer was used to measure the absorbance of the solution that was produced. A UV spectrophotometer was used to measure the absorbance at 593 nm, which allowed for the quantification of the ferrous tripyridyl triazine complex. With the help of ascorbic acid, a standard curve was created. (Maqsood et al., 2015) detailed the procedure that was followed to determine the activity, which was expressed as mg of ascorbic acid equivalents (AAE) per 0.1 kg of sample.

### 2.9 Machine learning and numerical modeling

The analysis of garlic processing data was conducted using four advanced machine-learning algorithms. Among these models, the support vector regressor (SVR) is a powerful technique commonly utilized for predictive modeling in machine learning (ML). The effectiveness of Support Vector Regression models in managing complex datasets has been demonstrated by their ability to navigate high-dimensional features and accurately capture non-linearity (H. Wang et al., 2023). SVRs utilize a spatial coordinate framework to distribute training samples, enhancing the differentiation between various sample categories. The objective is to identify a hyperplane that maximizes the margin while optimally distinguishing between different classes of samples. By identifying this hyperplane, SVRs can classify new samples based on which side of the divide they fall on, all within the same spatial domain. eXtreme Gradient Boosting (XGB) is a versatile, efficient, and portable library for distributed gradient boosting. The optimal settings for the model using XGB hyperparameter tuning alongside the GridSearchCV library are as follows: a learning rate of 0.05, 50 n estimators, and a subsample size of 0.3. This study also employed the Random Forest (RF) technique to simulate garlic storage patterns. A random forest enhances the bagging process by incorporating feature randomness and serves as a sophisticated meta-estimator. This method integrates multiple decision tree classifiers and regressors to improve predictive accuracy while reducing the risk of overfitting. The technique involves constructing several decision trees using different, randomly selected subsets of the dataset to maintain low correlation, followed by averaging their predictions. The optimized hyperparameters for the RF model are as follows: max_depth, min_samples_split, and n_estimators are all set to 10 (Zhang et al., 2024).

The storage mechanism for garlic was modeled using a recurrent neural network (RNN). Layers of interconnected nodes, or “artificial neurons,” make up artificial neural networks (ANNs), which are algorithms for computing. A synthetic neuron can receive signals from outside sources, analyze them appropriately, and then communicate with other neurons over a network. In an artificial neural network, the links between nodes, called edges, allow signals to be transmitted, much like synapses in a real brain. The weight of each connection changes as the network learns more. The size determines how strong the signal transmitted via the link is. Another interesting fact is that neurons can take a brink (El-Mesery, Qenawy, et al., 2024). A signal is sent out when the total amount of incoming signals exceeds this threshold. An artificial neural network's output is determined for each neuron by applying a non-linear. Because of this, the network can detect complex patterns and relationships in the data. In conclusion, ANNs can develop and learn from input data in a brain-like way because they mimic the functioning of biological neural networks through a network of linked artificial neurons. Using the GridsearchCV technique, we optimized several of the ANN's hyperparameters. After optimization, the hyperparameters were set to sigmoid for the first layer and tanh for the output layer. The first densely connected layer should have 15 units, the optimizer should have a learning rate of 0.01, and the dropout layer should have a rate of 0.5 (Chen, Zhang, et al., 2020).

### 2.10 Numerical optimization

Two sophisticated and effective optimization methods, Particle Swarm Optimization (PSO) and Differential Evolution (DE), were utilized to improve process conditions for optimizing self-life. Differential evolution is a powerful population-based search technique that performs exceptionally well in multi-modal settings and demonstrates significant effectiveness in the international optimization of intricate, nonlinear, and non-differentiable functions that are continuous. Its flexibility and reliability position it as an effective instrument for addressing optimization issues. Differential evolution (DE) functions iteratively, enhancing solutions via methods that effectively navigate the solution space. Such algorithms can efficiently explore extensive design spaces while making minimal or no assumptions regarding the fundamental optimization challenge (Cristina Stefania, 2021). In contrast to these frequently employed techniques, DE presents a unique benefit in effectively addressing nonlinear, non-differentiable problems, positioning it as a preferred option for complex optimization challenges where conventional methods may falter in attaining global convergence and optimization.

Conversely, particle swarm optimization (PSO) is a technique inspired by biological systems. This approach distinguishes itself from alternative methods by relying solely on the objective function without taking into account gradients or any other differential aspects of the objective. Metaheuristic PSO effectively navigates extensive solution spaces and addresses intricate optimization challenges with straightforward implementation and minimal domain expertise needed (G. Wang & Li, 2023). The algorithm is inspired by the natural behavior of bees as they gather in pursuit of food. A swarm is typically defined as a large group of simple, uniform entities that interact with their environment and one another in a localized way, governed by decentralized principles, leading to the emergence of behavior that has broader implications. In light of this, the flexibility of numerical experimentation has resulted in the widespread use of PSO for tackling various optimization issues (Gad, 2022). The flexibility of PSO allows it to tackle multiple real-world optimization problems in diverse fields, including engineering and computer science (Tam, 2021).

### 2.11 Principal component analysis (PCA)

PCA achieved dimensionality reduction, aiming to preserve the maximum variability within the dataset. The objective was to determine the primary factors affecting the variations in garlic's quality throughout the storage period. In preparation for PCA, the initial step involved standardizing the collected data to guarantee that all variables were aligned on a comparable scale, given their differences in units and magnitude. The covariance matrix was calculated using the standardized data to analyze the relationships among the variables. Calculating the eigenvalues and eigenvectors of the covariance matrix was performed to determine the principal components (PCs). Every component signifies a linear combination of the initial variables, with the first principal component (PC1) accounting for the most significant share of the variance within the dataset.

### 2.12 Statistical analysis

The results of each investigation were analyzed through analysis of variance (ANOVA) using the SPSS version 21.0 procedure, which was conducted in triplicate. Subsequently, we employed Duncan's Multiple Range Test to establish statistically significant means at the 5 % probability stage. The experimental data from the physiochemical study was meticulously gathered and organized, then transferred into Jupyter Notebook for further processing after being imported into a comprehensive spreadsheet. A one-hot encoding method was employed to convert the data, which included categorical variables such as storage temperature and packing material. Applying binary variables for each unique definite label converts categorical data into a numerical format. The data consisted of two primary categories: responses (the dependent variable) and forecasts (the independent variable). The predictors included storage temperature and packaging, while the response variables encompass firmness, weight loss, TSS, TA, pH, and moisture.

### 2.13 Data collection

This investigation involved storing garlic (*Allium sativum L.*) at 25 °C and 4 °C under controlled conditions, with various physicochemical variables measured over a specified duration. The study encompassed variables such as moisture content, weight loss, storage duration, TSS, TA, firmness, pH, and additional quality parameters. Data collection occurred consistently during storage, guaranteeing a thorough dataset for subsequent analysis.

## 3 Results and discussion

**Fig. 1:**
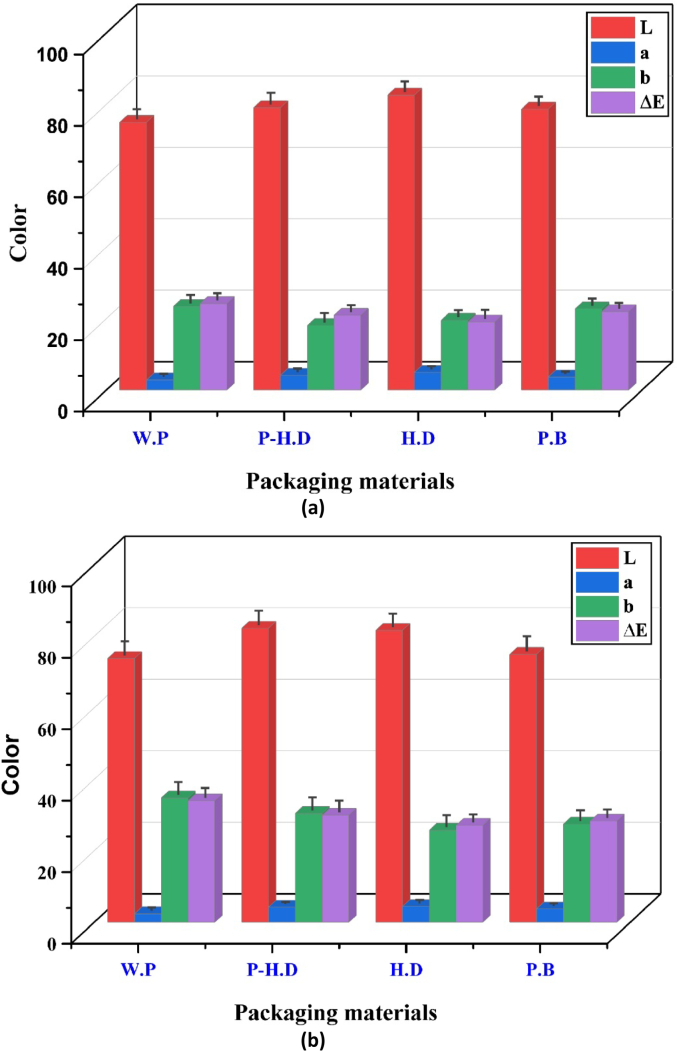
Changes in the color indices of garlic during the storage temperature and packaging materials: (a) Room temperature 25 °C; (b) Refrigerator 4 °C. L = lightness; a = redness–greenness; b = blue- ness–yellowness; ΔE = total color change; P-HD = Perforated High-Density Polyethylene; HD = High-Density Polyethylene; P·B = Paper Bags; W·P = Without Packaging. (For interpretation of the references to color in this figure legend, the reader is referred to the web version of this article.)

**Fig. 2:**
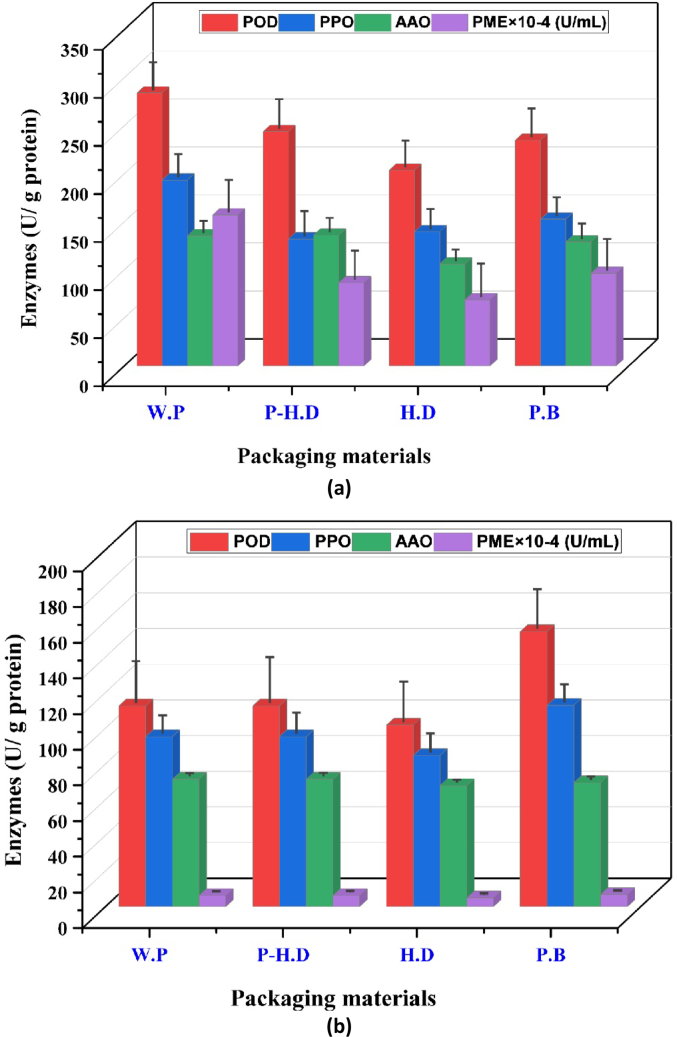
Changes in the enzyme activities of garlic during the storage temperature and packaging materials: (a) Room temperature 25 °C; (b) Refrigerator 4 °C. AAO = ascorbic acid oxidase; PPO = polyphenol oxidase; POD = peroxidase; PME = pectin methyl esterase; P-HD = Perforated High-Density Polyethylene; HD = High-Density Polyethylene; P·B = Paper Bags; W·P = Without Packaging.

**Fig. 3:**
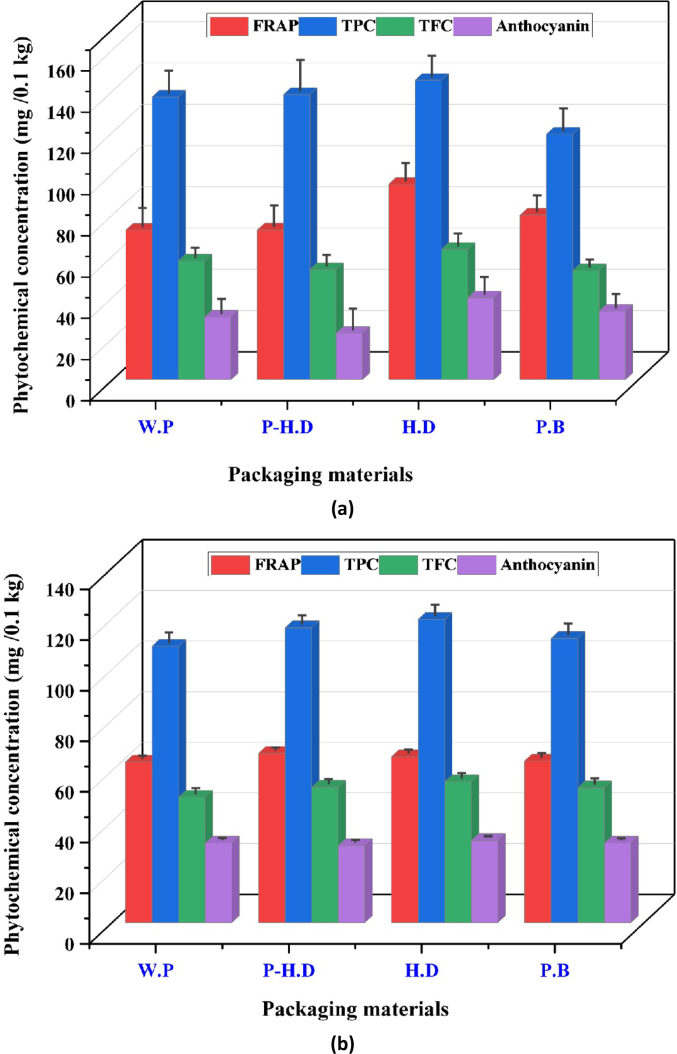
Changes in the phytochemical and antioxidant properties of garlic during the storage temperature and packaging materials: (a) Room temperature 25 °C; (b) Refrigerator 4 °C. FRAP = Ferric Reducing Antioxidant Power; TPC = total phenolic content; TFC = Total Flavonoid Content; ATC = Anthocyanin; P-HD = Perforated High-Density Polyethylene; HD = High-Density Polyethylene; P·B = Paper Bags; W·P = Without Packaging.

Figs. (1–3) show the research outcome on the color, activity of enzymes, phytochemical and antioxidant characteristics, packing conditions, and different packing products and their effects on garlic. Table 1, Table 2 summarize the outcomes of the (ML) models that examined the influence of storing circumstances and packaging bags on garlic's physical and chemical parameters. The data shows that the storage conditions and packing materials significantly affected garlic's color, enzyme activity, and phytochemical and antioxidant characteristics.

**Table 1:** Model performance and evaluation metrics for the different models.

Model Type	Loss Function	CV Training	CV Test	Prediction Training	Prediction Test
XGB	MSE	0.521	0.371	34.93	34.94
	RMSE	0.631	0.539	5.917	5.914
	MAE	0.515	0.457	18.51	18.52

SVR	MSE	0.480	0.415	34.814	34.91
	RMSE	0.651	0.594	5.90	5.90
	MAE	0.515	0.481	18.35	18.41

RF	MSE	0.462	0.387	34.84	34.85
	RMSE	0.589	0.511	5.951	5.90
	MAE	0.483	0.451	18.441	18.45

ANN	MSE	0.476	0.416	34.61	34.71
	RMSE	0.651	0.613	5.88	5.89
	MAE	0.523	0.465	18.23	18.31

XGB = eXtreme Gradient Boosting; SVR = Support Vector Regression; RF = random Forest; ANN = Artificial Neural Network; RMSE = Root Mean Square Error; MSE = Mean Square Error; MEA = Mean Absolute Error; CV = Cross-Validation.

**Table 2:** Machine learning-facilitated optimization of the garlic's physicochemical properties stored.

Model Type	Optimization Method	Packaging Materials	Storage Period	Firmness (N)	Weight Loss (%)	TSS (%)	TA (%)	pH	Moisture (%)
XGB	Diff Evolution	PB	1.36	11.61	5.16	34.75	0.98	5.95	61.85
XGB	Particle Swarm	WP	21.56	12.36	4.25	34.68	0.95	5.67	63.62
SVR	Diff Evolution	PB	0.00	14.10	1.31	34.52	1.25	4.86	63.89
SVR	Particle Swarm	HD	0.23	13.15	2.25	34.17	1.24	5.05	63.66
RF	Diff Evolution	HD	2.14	12.05	3.52	34.55	1.30	5.68	62.55
RF	Particle Swarm	PH	21.38	12.03	3.52	34.55	1.20	5.65	62.19
ANN	Diff Evolution	P-HD	0.06	13.44	1.47	34.51	1.20	4.76	63.66
ANN	Particle Swarm	P-HD	0.13	12.56	2.01	34.53	1.17	4.88	63.29

XGB = eXtreme Gradient Boosting; SVR = Support Vector Regression; RF = random Forest; ANN = Artificial Neural Network TSS = Total soluble solids; TA = Titratable acidity; P-HD = Perforated High-Density Polyethylene; HD = High-Density Polyethylene; P·B = Paper Bags; W·P = Without Packaging.

### 3.1 Color properties

Color is a crucial factor that influences the characteristics of garlic. Consequently, the parameters of lightness (L), blueness–yellowness (a), redness–greenness (b), and total color difference (δE) were assessed under various storage temperatures and across multiple packaging materials. The results demonstrated that both storage temperature and packaging material significantly affected garlic's color indices (Fig. 1). At 25 °C, unpackaged garlic exhibited the highest total color change (ΔE), reaching values up to 12.5 ± 0.8, indicating pronounced discoloration. In contrast, garlic stored at 4 °C in HD packaging showed minimal ΔE, with values around 4.2 ± 0.5, maintaining better visual quality. The lightness (L*) index was highest in HD-packaged garlic at 4 °C (78.6 ± 0.9) and lowest in unpackaged garlic at 25 °C (64.1 ± 1.2). The redness-greenness (a*) and yellowness-blueness (b*) parameters also shifted significantly, with a* values increasing up to 3.8 ± 0.3 at 25 °C (unpackaged) compared to 1.5 ± 0.2 at 4 °C (HD). These results underscore that cooler temperatures combined with protective packaging slow down undesirable color changes.

The results indicated notable influences of shelf-life duration, packaging type, and their relations to the color characteristics of the garlic. The specific kind of packaging bag exerted considerable influence on color characteristics, encompassing lightness, the balance of redness and greenness, blueness and yellowness, in addition to the overall color change of the garlic. Furthermore, the storage temperature exhibited a considerable impact, with a statistically significant effect (*p* < 0.05) noted across all color attributes, excluding lightness. Moreover, the interplay concerning storing conditions and packaging products was noteworthy solely for (a) and (b) at 5 %, suggesting that their combined effect impacted the color attributes associated with redness and yellowness. The noted variations in color characteristics highlight the necessity of choosing suitable packaging materials and enhancing storage conditions to maintain garlic's aesthetic appeal. This information provides a significant framework for improving the quality and marketability of garlic throughout its storage and distribution processes.

Fig. 1 (a,b) further illustrates the variations in the average color characteristics of the garlic subjected to various storage temperatures. The maximum (L) was noted in HD at 4 °C, while the minimum values were seen in unpackaged garlic at 25 °C. The findings additionally indicated elevated values of (b) and (δE) corresponding to higher temperatures. The most elevated (b) and (δE) values were observed in garlic without packaging (WP) at 25 °C, while the lowest values were noted across all samples throughout the storage period at 4 °C. Additionally, the highest mean values of (L) and (a) were recorded in color attributes, and the garlic's enzyme activities significantly increased with rising storage temperatures (Park et al., 2019). The highest mean enzyme activities were observed in unpackaged garlic and those contained in Perforated High-Density Polyethylene (P-HD)and paper bags (PB) at 25 °C. Cold storage temperature has been demonstrated to reduce PPO activity within samples, though it does not entirely stop it (Jung et al., 2019). The highest mean PPO content was observed in the no-packaging (WP) samples and those containing garlic packed in P-HD at 25 °C.

### 3.2 Enzyme activities

Enzyme activities were notably influenced by storage conditions (Fig. 2). The highest peroxidase (POD) activity was recorded in unpackaged garlic at 25 °C, peaking at 18.5 ± 1.1 U/g protein, while the lowest was observed in HD-packaged garlic at 4 °C (7.2 ± 0.8 U/g protein). Similarly, PPO activity increased significantly at higher temperatures, with unpackaged garlic reaching 14.3 ± 1.0 U/g protein at 25 °C versus 6.0 ± 0.7 U/g protein at 4 °C. Ascorbic acid oxidase (AAO) activity followed the same pattern, rising to 12.7 ± 0.9 U/g protein in P-HD packaging at 25 °C, while remaining low (5.5 ± 0.6 U/g protein) in HD packaging at 4 °C. The results clearly demonstrate that elevated storage temperature accelerates enzymatic activities associated with browning and quality degradation, emphasizing the benefit of cold storage combined with appropriate packaging.

Therefore, storage conditions and packing bags may modulate enzyme activity in samples, affecting shelf life and attributes. The findings demonstrate that both the temperature of storage condition and the packaging type are crucial factors affecting the enzyme activities of fresh garlic. Temperature fluctuations can either speed up or slow down enzymatic reactions; therefore, meticulous management of storage conditions is crucial for preserving quality. The selection of packaging material holds significant importance, as it impacts elements like gas exchange, moisture content, and safeguarding against external factors. This research highlights the importance of choosing suitable packaging materials that align with the unique enzymatic characteristics of garlic. Moreover, the interplay between storage conditions and packaging material highlights the intricacies of maintaining enzyme activities. It indicates that considering both factors, a cohesive approach is crucial for enhancing storage strategies. The results of this research offer a significant understanding of the agricultural and food sectors, allowing for the adoption of efficient storage methods that maintain the enzymatic characteristics and quality of fresh garlic, thereby enhancing the quality of produce available to consumers. Fig. 2 (a, b) illustrates that the average enzyme activities of garlic, including POD, PPO, AAO, and PME, significantly increased with rising storage temperatures. The findings demonstrate that both the temperature of storage condition and the packaging type are crucial factors affecting the enzyme activities of fresh garlic. Garlic that was unpackaged and those contained in P-HD and (PB) at 25 °C exhibited the highest average enzyme activities (El-Mesery, Adelusi, et al., 2024). The highest average values of POD content were observed in the no-packaging samples and those packed in PHDP at 25 °C.

### 3.3 Phytochemical and antioxidant properties

An analysis of variance (ANOVA) was performed to evaluate the effects of storage duration and packaging material on the phytochemical and antioxidant properties of fresh garlic, including FRAP, TPC, TFC, and Anthocyanin. The findings in Fig. 3(a) and (b) demonstrate notably significant effects across all factors examined. The storage temperature exhibits a notable and considerable impact (*p* < 0.05) on all measured phytochemical and antioxidant properties except for anthocyanin. Phytochemical retention and antioxidant capacity were significantly affected by storage variables (Fig. 3). Total phenolic content (TPC) decreased markedly at 25 °C, with unpackaged garlic dropping to 42.5 ± 2.3 mg GAE/100 g, compared to 67.8 ± 2.0 mg GAE/100 g in HD packaging at 4 °C. Similarly, FRAP values, an indicator of antioxidant potential, were highest at 4 °C in HD packaging (5.2 ± 0.3 mg AAE/100 g) and lowest in unpackaged garlic at 25 °C (2.4 ± 0.2 mg AAE/100 g). Total flavonoid content (TFC) and anthocyanin levels also showed significantly greater retention at lower temperatures, confirming that cold storage and protective packaging help maintain garlic's bioactive compounds.

Furthermore, the packaging material and the interaction effect of storage temperature and packaging materials significantly influenced the phytochemical and antioxidant content of the garlic. The findings highlight the significance of storage conditions and packaging materials in preserving the phytochemical and antioxidant properties of fresh garlic. Temperature variations significantly impacted the antioxidant capacity and phytochemical content, emphasizing the importance of meticulous temperature control during storage. The selection of packaging material proved to be a vital element, as it had a significant impact on all assessed properties. This highlights the importance of selecting suitable packaging materials that can accommodate garlic's unique phytochemical and antioxidant properties.

Furthermore, the complexities of preserving these characteristics are further illustrated by the correlation between storage circumstances and packaging material (Esua et al., 2019). An innovative study conducted by (Mir et al., 2015) the TPC content was highest in candy that was wrapped in laminates, then in plastic jars, and finally in polyethylene. The degradation of FRAP and anthocyanin in tomatoes was influenced by higher temperatures and the type of packing material (EL-Mesery et al., 2022). In quince toffy, a similar relationship between storage temperature and FRAP was found (Mir et al., 2015). This suggests that a comprehensive approach is essential for optimizing storage strategies, taking into account both factors. The results of this research offer significant implications for the agricultural and food sectors. They enable the adoption of efficient storage methods that preserve the phytochemical and antioxidant qualities of fresh garlic, thereby enhancing the quality of produce available to consumers.

### 3.4 Predictive modeling of storage conditions

Four distinct machine-learning algorithms were generalized using a large body of experimental data on the drying of fresh samples. The models used an iterative algorithm to train their loss functions with the lowest possible root-mean-square error (RMSE). A lower loss function value indicates a better fit, whereas a value of 0 indicates a perfect fit, showing that the projected values align more with the actual values. The performance and learning aspects of the models have to be evaluated by carefully comparing the projected and actual values using the mean-square error (MSE), RMSE, mean absolute error (MAE), and R^2^. Table 1 shows that although all models fit the data relatively well, the models' performance differed according to the evaluation metric and response variable. As an alternative, an R^2^ value of 1 denotes a perfect fit, and a higher R^2^ value strengthens the model's prediction performance.

**Fig. 4:**
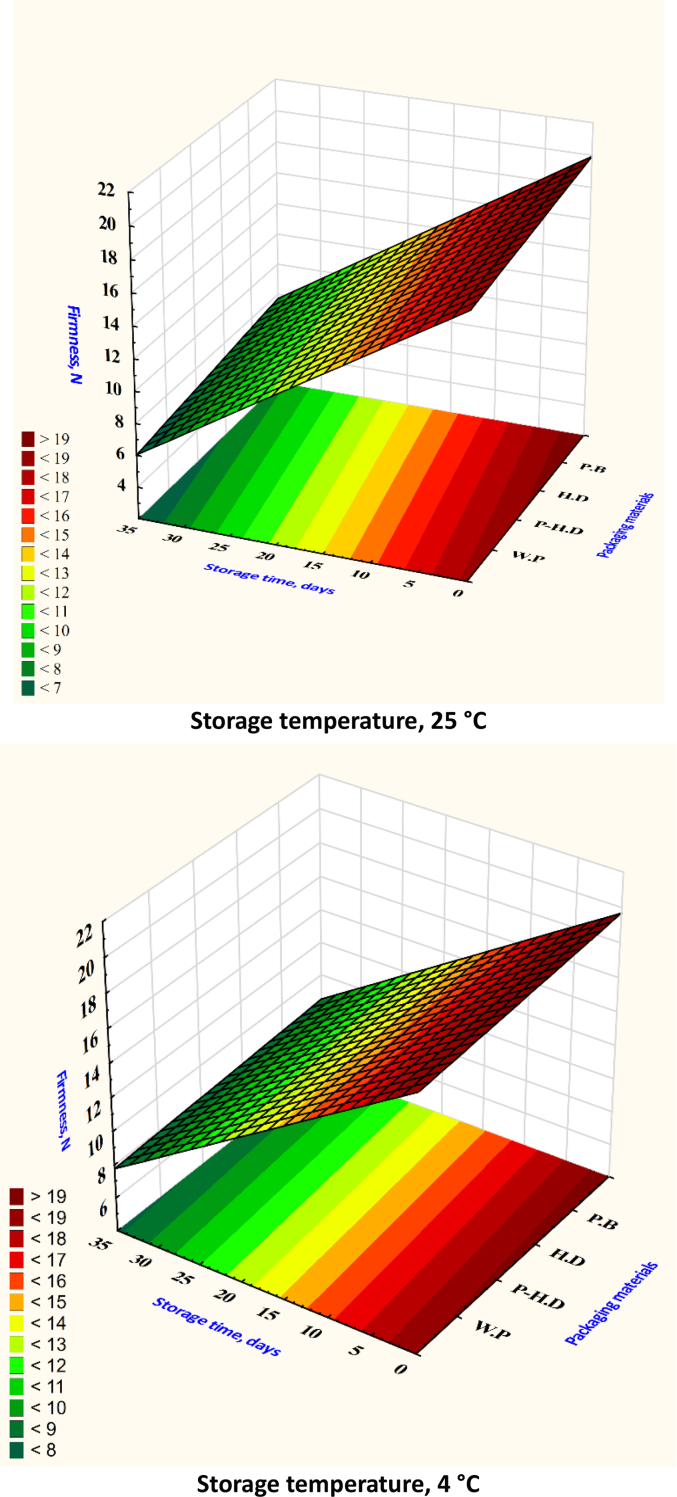
Changes in garlic's firmness during the storage time under different storage temperatures and packaging materials. P-HD = Perforated High-Density Polyethylene; HD = High-Density Polyethylene; P·B = Paper Bags; W·P = Without Packaging.

**Fig. 5:**
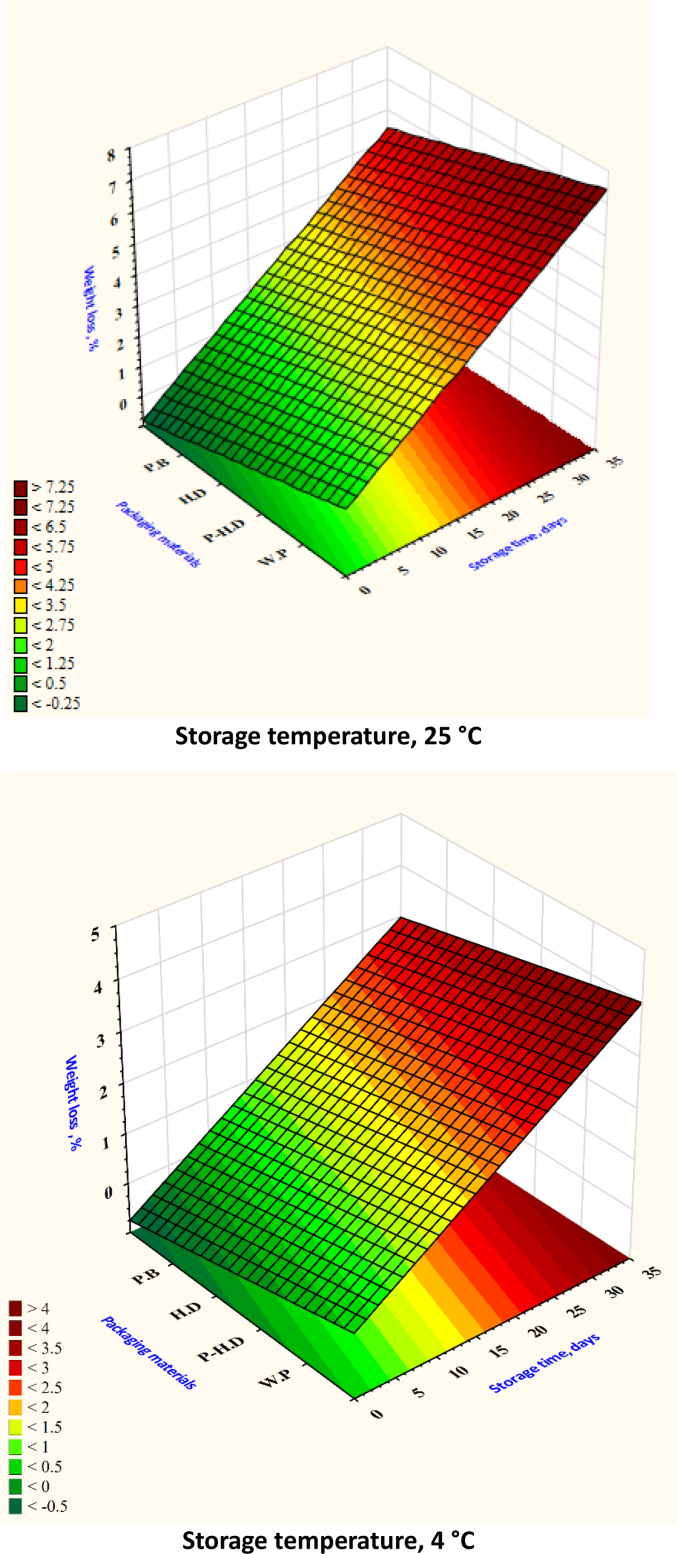
Changes in garlic's weight loss during the storage time under different storage temperatures and packaging materials. P-HD = Perforated High-Density Polyethylene; HD = High-Density Polyethylene; P·B = Paper Bags; W·P = Without Packaging.

**Fig. 6:**
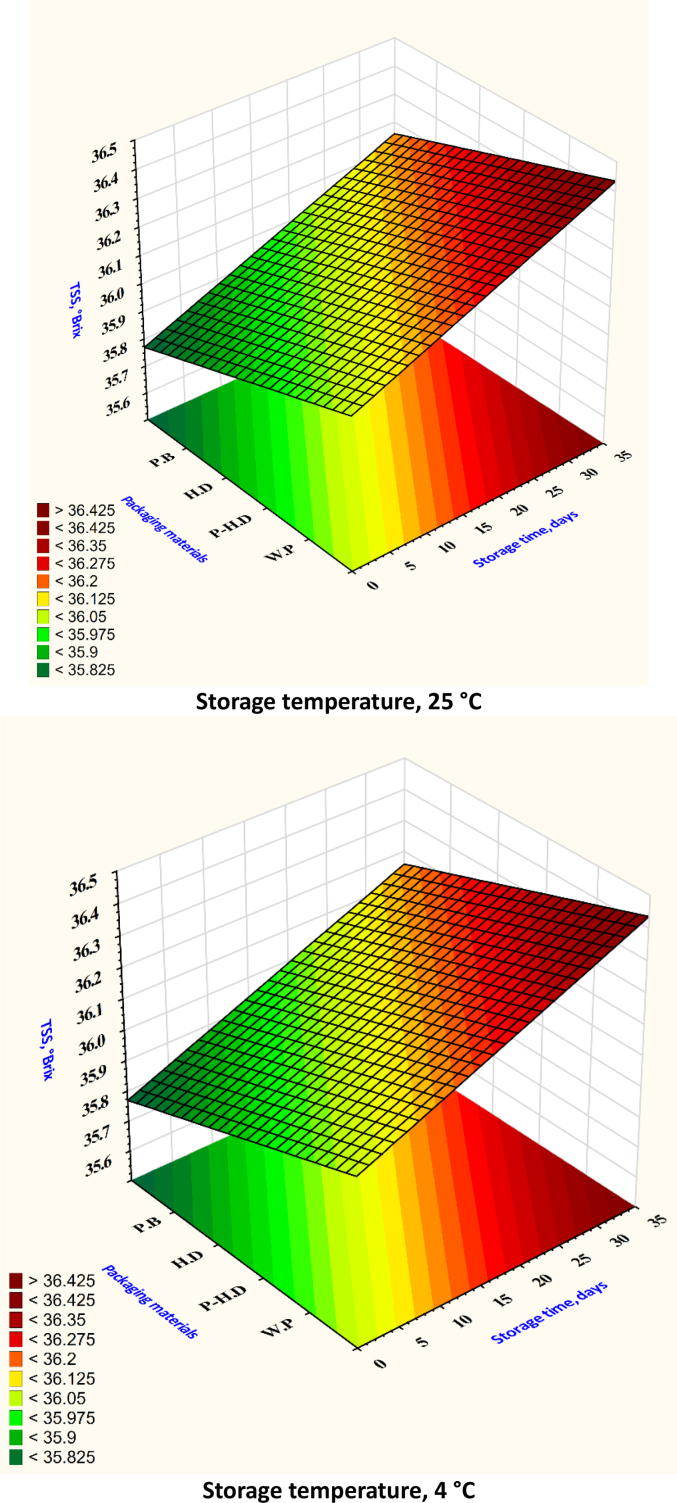
Changes in garlic's TSS during the storage time under different storage temperatures and packaging materials. P-HD = Perforated High-Density Polyethylene; HD = High-Density Polyethylene; P·B = Paper Bags; W·P = Without Packaging.

**Fig. 7:**
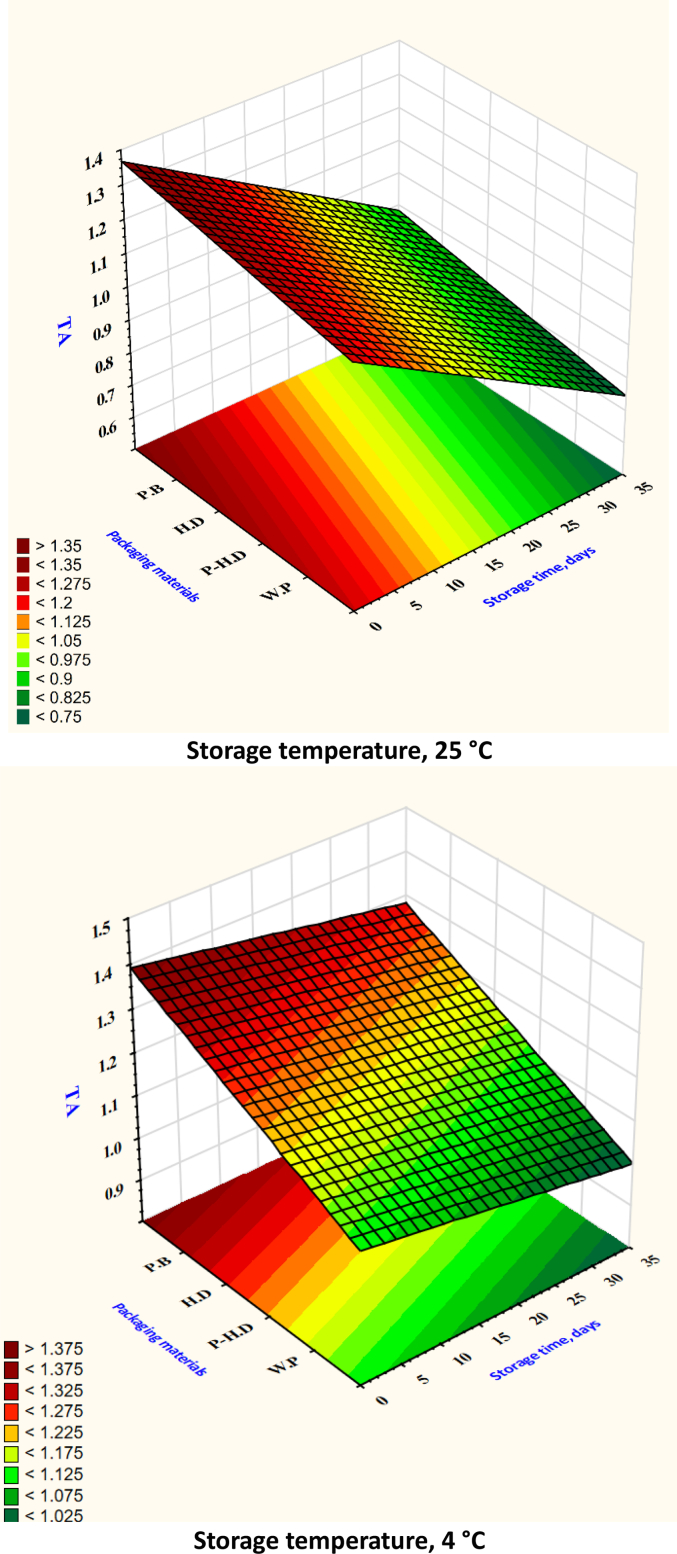
Changes in garlic's TA during the storage time under different storage temperatures and packaging materials. P-HD = Perforated High-Density Polyethylene; HD = High-Density Polyethylene; P·B = Paper Bags; W·P = Without Packaging.

**Fig. 8:**
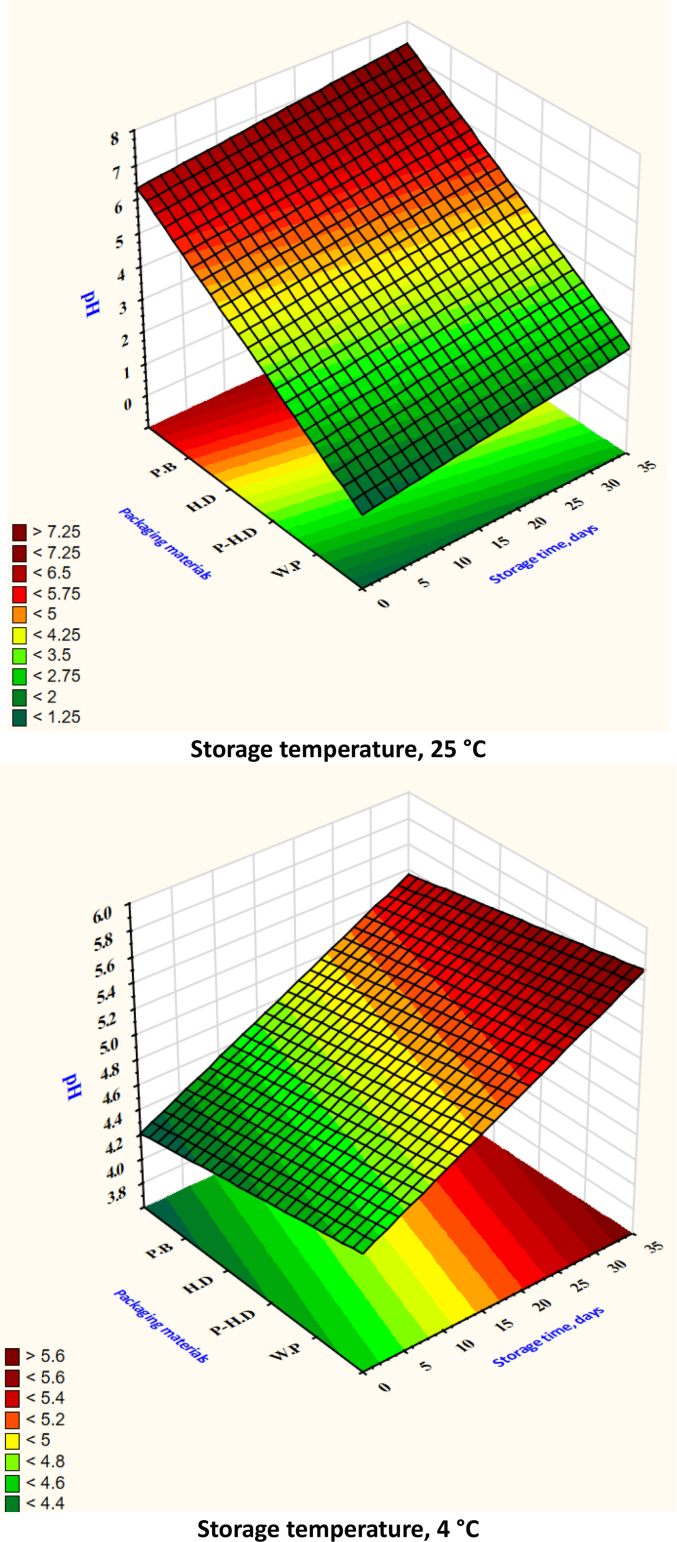
Changes in garlic's pH during the storage time under different storage temperatures and packaging materials. P-HD = Perforated High-Density Polyethylene; HD = High-Density Polyethylene; P·B = Paper Bags; W·P = Without Packaging.

**Fig. 9:**
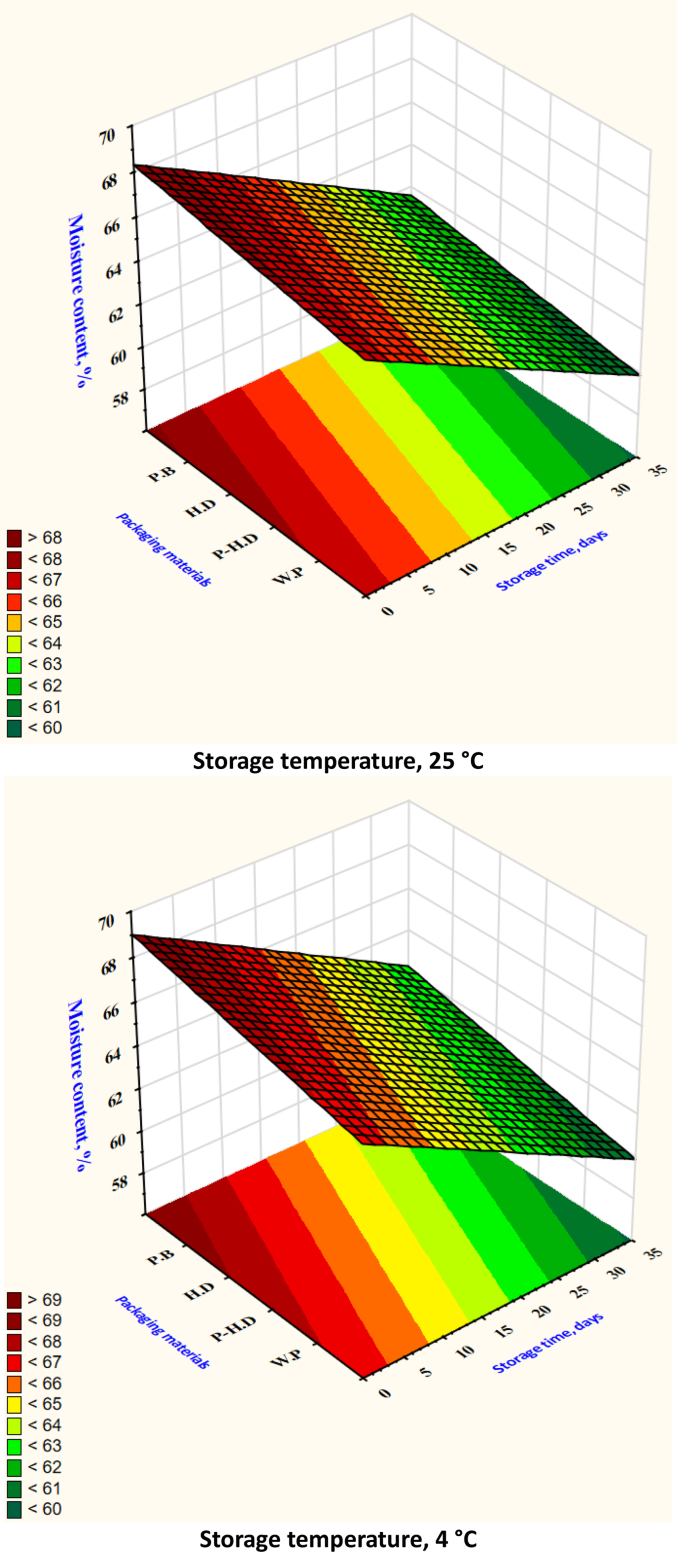
Changes in garlic's Moisture content during the storage time under different storage temperatures and packaging materials. P-HD = Perforated High-Density Polyethylene; HD = High-Density Polyethylene; P·B = Paper Bags; W·P = Without Packaging.

After analyzing the test set through cross-validation, it was found that the XGB model had the best MSE and RMSE. Table 1 shows that the ANN model obtained the best values for MAE, RMSE, and MSE in the prediction set. When comparing average MSE, RMSE, and MAE, the ANN model fared better than the RF and SVR models. Based on the R^2^ values, the RF model emerged as the top predictor of firmness (Fig. 4), weight loss (Fig. 5), TSS (Fig. 6), TA (Fig. 7), pH (Fig. 8), and moisture content (Fig. 9). Firmness decreased over storage time, with a sharper decline at 25 °C (Fig. 4). By the end of storage, garlic firmness in unpackaged samples at 25 °C dropped to 6.5 ± 0.4 N, while HD-packaged garlic at 4 °C retained firmness at 12.3 ± 0.6 N, almost double the firmness of the 25 °C sample. The Weight loss was highest in unpackaged garlic at 25 °C, reaching 14.2 % ± 0.5 after 30 days, compared to only 5.8 % ± 0.4 in HD-packaged garlic at 4 °C (Fig. 5). This highlights the critical role of both temperature and packaging in limiting dehydration and mass loss during storage.gs emphasize that cold storage conditions significantly slow softening and preserve texture.

Total soluble solids (TSS) showed a declining trend at higher temperatures. Garlic stored at 25 °C without packaging dropped to 2.9 % ± 0.2, while garlic at 4 °C in HD packaging maintained TSS around 4.6 % ± 0.3 (Fig. 6). Titratable acidity (TA) remained relatively stable, though slight decreases were noted at higher temperatures: unpackaged garlic at 25 °C showed a TA of 0.85 % ± 0.05 compared to 1.20 % ± 0.04 at 4 °C (Fig. 7). pH values increased at 25 °C, reflecting a loss of acidity, rising from 5.8 ± 0.1 to 6.4 ± 0.1 (Fig. 8), while remaining around 5.9 ± 0.1 in HD-packaged garlic at 4 °C. Moisture retention was best in HD packaging at 4 °C (65.4 % ± 1.2) and lowest in unpackaged garlic at 25 °C (53.2 % ± 1.3) (Fig. 9). The data reinforce the importance of combining low temperature with effective packaging to prevent excessive water loss.

Additionally, the models accurately characterized the variation in stiffness. The pH showed the most extreme non-systematic variance, with values that changed by the greatest. Assuming the assessment measures for the two sets of data are quite similar, the model is functioning effectively and exhibits outstanding learning characteristics. The models' low MSE, RMSE, and MAE values (Fig. S1-S6) demonstrate their excellent accuracy and efficacy as forecasting tools.

A strong statistical method, the 5 × 2 design, was used to evaluate the effectiveness of two models by cross-validating the data. This approach was employed to assess the relative performance of each model in a paired comparison: the 5 × 2 cross-validated paired *t*-test. We split the dataset in half and utilized one half for training and the other half for testing. We do this five times. Two subsets are used for training each model in each iteration, whereas three subsets are used for testing. The following step is to use these portions for training and testing the models (Dong et al., 2017). This provides a fair opportunity for both models to train and be assessed for every data segment. We then use a paired t-test to compare the performance characteristics of the iterations. The *p*-value will indicate whether there is a significant difference in the model's performance. Neither the resampled paired t-test nor the k-fold cross-validated paired t-test can compare to the superiority of this method. This study found that the models' performances varied substantially, as indicated by a 5 × 2 Cross-Validation. (CV) paired t-test. A notable disparity in performance was observed between the ANN and RF models, as indicated by the results (*p* < 0.05). There was a significant difference (p < 0.05) between the XGB model's performance and that of the ANN model. The XGB, SVR, and RF variants did not vary significantly in terms of performance, although the difference was not statistically significant (*p* > 0.05). Using the metrics (MSE, RMSE, and MAE), you can sort the models in order of effectiveness. The sequence is XGB, followed by ANN, RF, and then SVR.

### 3.5 Machine learning optimization

The developed models were employed alongside PSO and differential evolution (DE) to determine the optimal conditions for drying garlic in the solar dryer. The optimal conditions outlined here signify the most effective storage parameters for ensuring the highest quality of the product (Table 2). The findings from all four machine learning models indicate that the ideal storage conditions for the product are 4 °C, surpassing the conditions at 25 °C. Furthermore, most models indicate that following the drying and storage processes, items produced with (HD) packaging material generally exhibit exceptional quality. According to the optimal circumstances identified by the RF model through the particle swarm optimization method, the quality of the product can be expected to match that of products stored for under 24 h when maintained at 4 °C, packaged in HD, and stored for 21 days (Table 2). An analogous estimation was conducted employing the XGB-particle swarm optimization technique (Bai et al., 2018).

### 3.6 Principal component analysis (PCA)

**Fig. 10:**
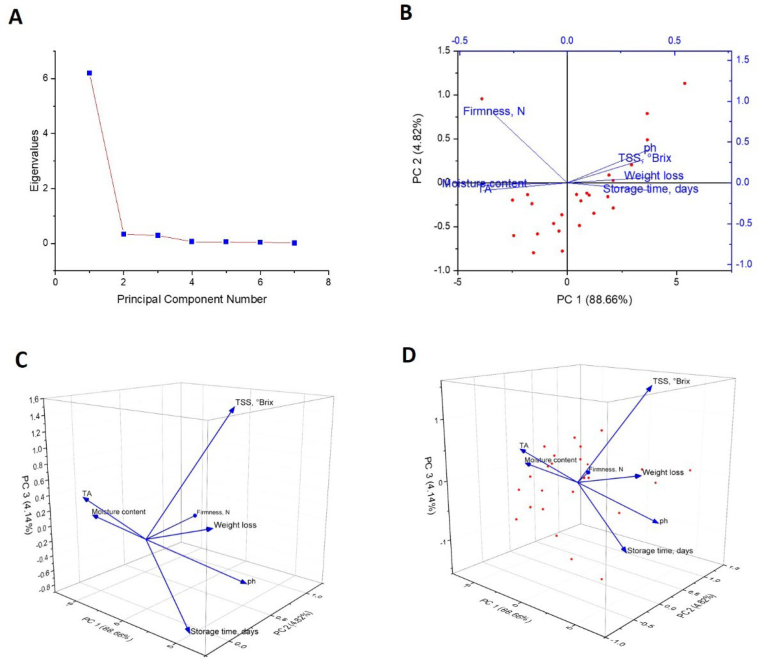
Principal Component Analysis (PCA) of physicochemical parameters and phytochemicals of stored garlic under varied storage packaging at a storage temperature of 25 °C.

The Principal Component Analysis (PCA) results depicted in Fig. 10 provide significant insights into how storage conditions affect garlic's physicochemical and phytochemical compositions (*Allium Sativum L.*) when stored at 25 °C. The findings provide an in-depth analysis of the elements influencing garlic's quality throughout storage, presenting practical recommendations for enhancing storage environments and packaging solutions. The scree plot (Fig. 10A) illustrates the eigenvalues corresponding to each principal component (PC). The initial principal component (PC1) encompasses the most significant portion of the variance within the dataset, representing 88.66 %. This indicates that PC1 plays a crucial role in affecting the physicochemical properties of garlic during storage, while the second principal component (PC2) contributes merely 8.62 % to the overall variance. The significant decline in eigenvalues following the first principal component suggests that later components contribute little, reinforcing the critical role of PC1 in accounting for most of the observed variation.

The 2D biplot (Fig. 10) demonstrates the connections among essential variables (e.g., moisture content, storage time, weight loss, TSS, and Brix) and the initial two principal components. The analysis reveals that factors like moisture content and storage duration exhibit robust correlations with PC1 and PC2, highlighting their crucial influence on the variations in garlic quality as time progresses. This finding is consistent with earlier studies that highlight the significance of moisture content in preserving the quality of stored food products (Alonso-Salces et al., 2005) The observed separation in the plot indicates that varying storage conditions can significantly affect garlic's physicochemical properties, highlighting the importance of environmental factors, such as moisture levels, during storage.

The 3D plots (Fig. 10C and D) comprehensively show the interconnections among the variables and principal components. The visualizations indicate that moisture content and TSS play significant roles in determining the quality of garlic. The relationship between moisture content and weight loss is particularly important, reinforcing the conclusions of (Rui Zhao et al., 2020), which indicated that moisture loss hastens the decline in garlic quality during storage. The findings align with earlier research, demonstrating that the relationship between moisture content and storage duration aligns with the study conducted by (F. Chen & Rohe, 2024), which emphasized the significance of moisture content in determining garlic's shelf life and quality. In a similar vein, TSS and Brix's critical influence on garlic's shelf life is corroborated by (Mancini et al., 2017), who discovered that these parameters serve as indicators of garlic's sweetness and overall quality, both of which typically diminish over time due to metabolic breakdown. The results of this PCA study offer valuable insights for enhancing garlic storage and packaging methods. As demonstrated by the PCA results, producers can create more efficient packaging materials that extend shelf life and preserve the quality of garlic by optimizing moisture content and reducing weight loss. This aligns with previous investigations, including those by (Mancini et al., 2017), which highlighted the significance of appropriate packaging in minimizing moisture loss and prolonging the shelf life of agricultural products. Moreover, combining these PCA results with artificial intelligence (AI) and machine learning (ML) models presents a promising strategy for forecasting the physicochemical changes of garlic under different storage conditions. Utilizing AI and ML models can optimize storage parameters and packaging materials, improving quality control across the supply chain. This method has the potential to greatly minimize food waste and enhance the sustainability of garlic production, which holds considerable importance for the agricultural sector. Utilizing these insights with AI and ML models allows the farm sector to improve predictions and management of garlic shelf life, bolster food security, and minimize waste.

**Fig. 11:**
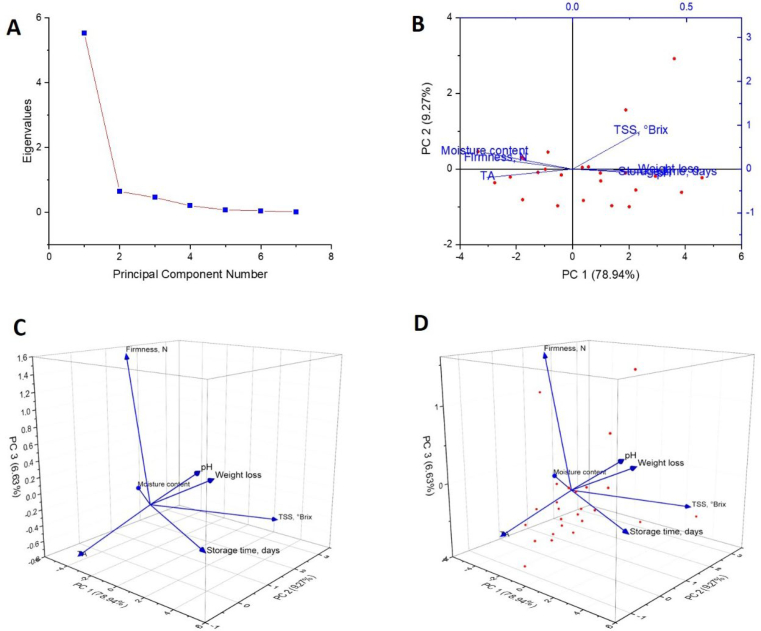
Principal Component Analysis (PCA) of physicochemical parameters and phytochemicals of stored garlic under varied storage packaging at a storage temperature of 4 °C.

The Principal Component Analysis (PCA) findings on garlic stored at 4 °C offer essential insights into the fundamental factors influencing garlic's physicochemical and phytochemical characteristics throughout the storage period. The results provide an in-depth examination of the impact of different storage conditions on the quality of garlic, particularly emphasizing the significant effect of temperature in extending its shelf life. The scree plot (Fig. 11A) shows that PC1 explains 78.94 % of the total variance, suggesting that this component captures most of the data's variability. The findings indicate that the first principal component predominantly accounts for the variations in garlic's physicochemical properties when stored at 4 °C. The results at 4 °C highlight the prominence of PC1, reinforcing the stability of garlic's physicochemical composition at lower temperatures, with only slight degradation observed over time when compared to the findings at 25 °C. PC2 accounts for 9.27 % of the variance, indicating secondary factors influencing the data; however, its contribution is relatively minor. The significant decline in eigenvalues following PC1 suggests that storage temperature is crucial in determining garlic quality, surpassing other factors' influence.

The biplot (Fig. 11B) illustrates the influence of essential factors like moisture content, TSS, storage time, and weight loss on the variability represented by PC1 and PC2. The significant relationship between moisture content and storage time with PC1 highlights their essential contributions to preserving garlic quality. The retention of moisture is a recognized element that affects the maintenance of garlic's nutritional and sensory characteristics, especially during cold storage. The results at 25 °C exhibited more significant variability in weight loss and changes in TSS, whereas at 4 °C, the garlic demonstrated less variation. This suggests that cold storage effectively reduces the rate of metabolic processes, including respiration and moisture loss. This is consistent with the findings of (Chen, Zhu, et al., 2020), which emphasized that preserving a higher moisture content during storage is crucial for prolonging shelf life.

The 3D plots (Fig. 11C and D) illustrate the complex relationships among the physicochemical properties and their corresponding principal components. The evident correlation between moisture content and weight loss is observable, as both factors significantly impact the overall variance represented by PC1. This correlation aligns with previous findings by (Renjie Zhao et al., 2022), which showed that moisture loss directly affects the degradation of garlic quality. With extended storage duration, there is a reduction in moisture content, resulting in increased weight loss and possible shrinkage, ultimately affecting the shelf life of garlic. The decreased degradation observed at 4 °C is due to diminished metabolic activity, which hinders the enzymatic reactions that lead to quality decline. When analyzing these results alongside the data collected at 25 °C, it becomes evident that reduced temperatures significantly enhance the preservation of garlic's quality over longer durations. Storing at 4 °C leads to diminished weight loss and a more gradual decline in TSS, emphasizing the advantages of cold storage. The results align with the observations made by (Mancini et al., 2017), who indicated that garlic kept at lower temperatures preserves superior texture, flavor, and nutritional quality in contrast to garlic stored at elevated temperatures. The lowered metabolic rate at 4 °C restricts the enzymatic degradation of sugars, starches, and water-soluble compounds, thereby allowing garlic to preserve its intended quality for extended durations. The relationship between moisture content and weight loss identified in the PCA results highlights the significance of managing moisture during storage, especially at reduced temperatures. Prior investigations, such as those conducted by (Mancini et al., 2017), have reliably demonstrated that managing moisture loss during storage can greatly prolong the shelf life of garlic and various other vegetables, especially in cold storage environments. The significance of these findings lies in their highlighting the necessity for efficient packaging solutions that reduce moisture loss and inhibit degradation over time.

The findings from this PCA study indicate that maintaining a storage temperature of 4 °C is ideal for preserving the quality of garlic. The combined effect of packaging and storage temperature was evident across all parameters. HD and P-HD packaging at 4 °C consistently preserved garlic's physicochemical, phytochemical, and textural properties better than other combinations. PCA results further confirmed that moisture content and storage duration were the principal factors driving variance in garlic quality, accounting for approximately 88 % of variability at 25 °C and estimated 79 % at 4 °C (Fig. 10, Fig. 11). This demonstrates that a synergistic strategy is essential; temperature and packaging must be optimized together to achieve the best storage outcomes. The results emphasize the importance of effective moisture management and precise regulation of storage duration to preserve garlic's physicochemical and phytochemical characteristics. Furthermore, these findings establish a solid foundation for incorporating AI and machine learning models to forecast the quality of garlic over time across different storage conditions. These models can be crucial in developing more effective storage strategies and packaging materials that enhance shelf life and reduce food waste. The PCA findings for garlic stored at 4 °C indicate that moisture content and storage duration are the primary factors affecting preserving garlic's quality. Employing cold storage markedly diminishes the deterioration of garlic's physicochemical characteristics, rendering it exceptionally efficient for prolonging shelf life. The agricultural sector can use AI and machine learning models to enhance storage practices, minimize waste, and refine packaging solutions to improve garlic preservation. The results of this study advance the overarching aim of enhancing food security, minimizing spoilage, and fostering sustainable practices within the agricultural sector.

## 4 Conclusion

Integrating machine learning models with AI-assisted optimization has excellent potential to improve garlic postharvest management procedures. By delving into the intricate web of relationships between storage conditions, packing materials, and garlic qualities, this area of inquiry has the potential to revolutionize the garlic supply chain, providing customers with garlic that is healthier, longer-lasting, and of higher quality. The combined effect of packaging and storage temperature was evident across all parameters. HD and P-HD packaging at 4 °C consistently preserved garlic's physicochemical, phytochemical, and textural properties better than other combinations. PCA results further confirmed that moisture content and storage duration were the principal factors driving variance in garlic quality, accounting for approximately 88 % of variability at 25 °C and estimated 79 % at 4 °C. This demonstrates that a synergistic strategy is essential; temperature and packaging must be optimized together to achieve the best storage outcomes. To maintain garlic's freshness and nutritional value after harvest, storing it in the best possible circumstances and using the right packaging materials is essential. New research and development in this field bode well for reducing food waste, increasing food security, and catering to customers' demands for longer-lasting, healthier garlic products. The postharvest process relies heavily on understanding and enhancing storage conditions and packaging tactics. These programs help farmers and distributors out by reducing food waste and ensuring that people buy garlic that is both healthy and delicious.

However, this study has certain limitations. First, the model's predictions are based on specific environmental and storage parameters and may not fully capture the variability across different geographic regions, garlic varieties, or unforeseen storage conditions. Additionally, while the study focused on select packaging materials and storage factors, other influential variables such as microbial activity, transportation dynamics, and consumer handling practices were not considered.

Future research should aim to validate these findings under diverse real-world conditions, explore the integration of additional environmental and biological variables, and assess the economic feasibility of implementing AI-assisted optimization at larger scales. Expanding the study across different regions and garlic cultivars would further strengthen the generalizability and practical application of the results.

## CRediT authorship contribution statement

**Hany S. El-Mesery:** Writing – review & editing, Writing – original draft, Validation, Software, Methodology, Investigation, Formal analysis, Data curation, Conceptualization. **Ahmed H. ElMesiry:** Writing – review & editing, Validation, Software, Formal analysis, Conceptualization. **Mansuur Husein:** Resources, Investigation, Data curation. **Zicheng Hu:** Writing – review & editing, Supervision, Resources. **Ali Salem:** Writing – review & editing, Resources, Investigation, Funding acquisition, Data curation.

## Declaration of competing interest

The authors declare that they have no known competing financial interests or personal relationships that could have appeared to influence the work reported in this paper.

## Data Availability

Data will be made available on request.

## Appendix A Supplementary data

Supplementary material Image 1
