# Supplementary material for: Artificial intelligence and machine learning models for predicting and evaluating the influence of shelf-life environments and packaging materials on garlic (Allium Sativum L) physicochemical and phytochemical compositions
Source: Food Chem X. 2025 Jul 6;29:102731. doi: 10.1016/j.fochx.2025.102731 (PMC12274686; doi:10.1016/j.fochx.2025.102731)
Supplement: Supplementary file 1 — Supplementary material [file mmc1.docx]

| 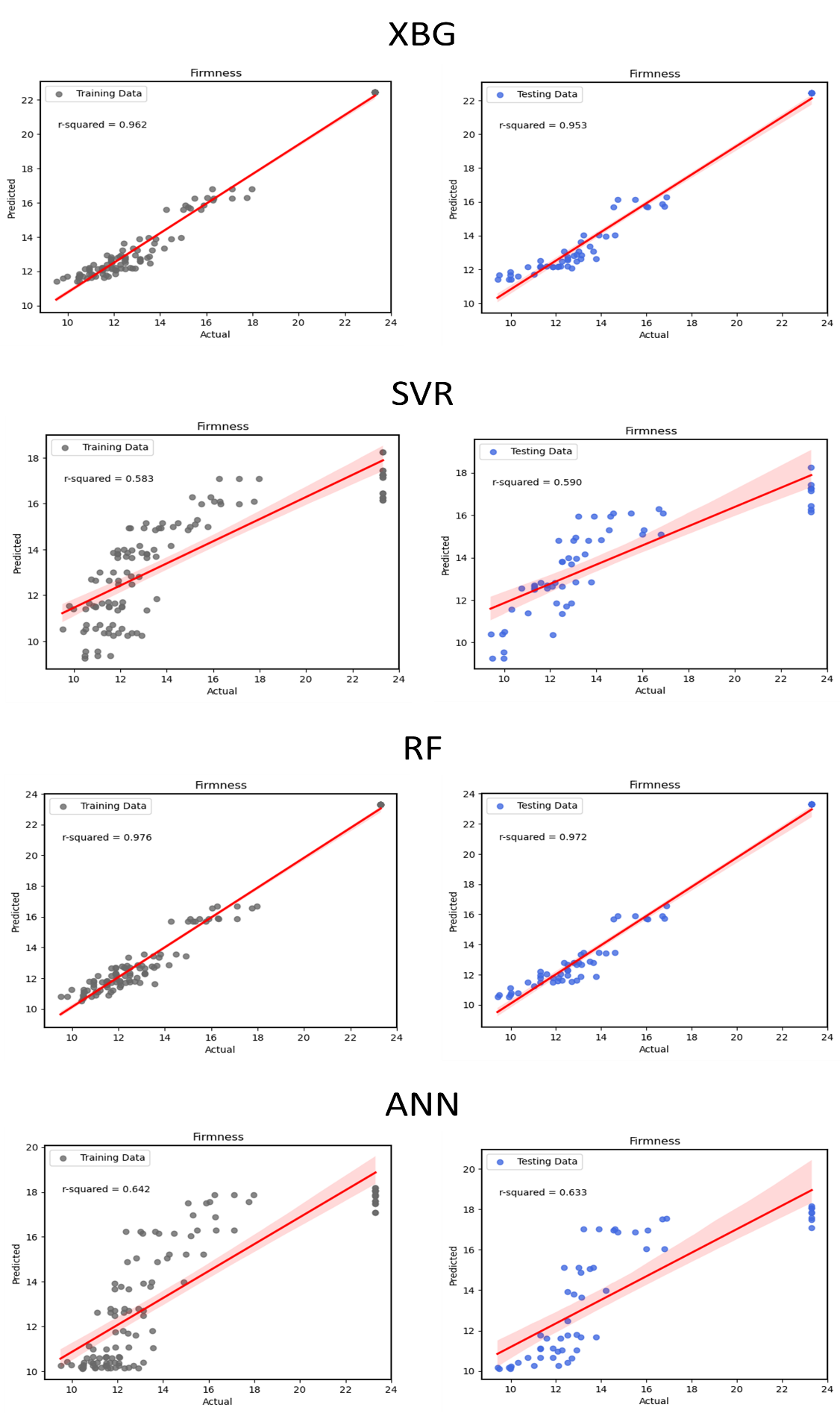 |
| --- |
| Fig. S1. Comparison between experimental data for garlic's firmness and the predicted values by different ML techniques. |
| 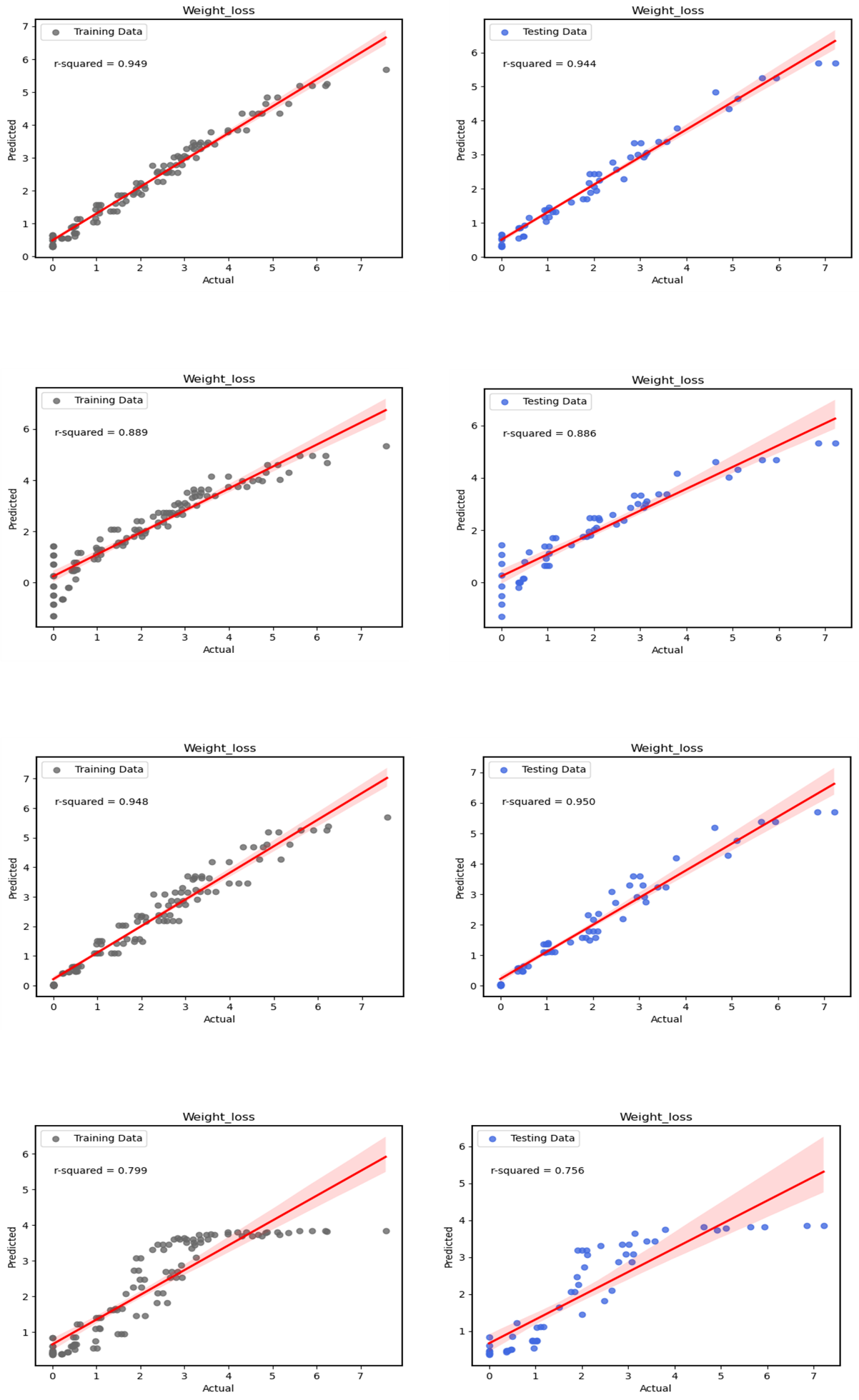 |
| Fig. S2. Comparison between experimental data for garlic's weight loss and the predicted values by different ML techniques. |
| 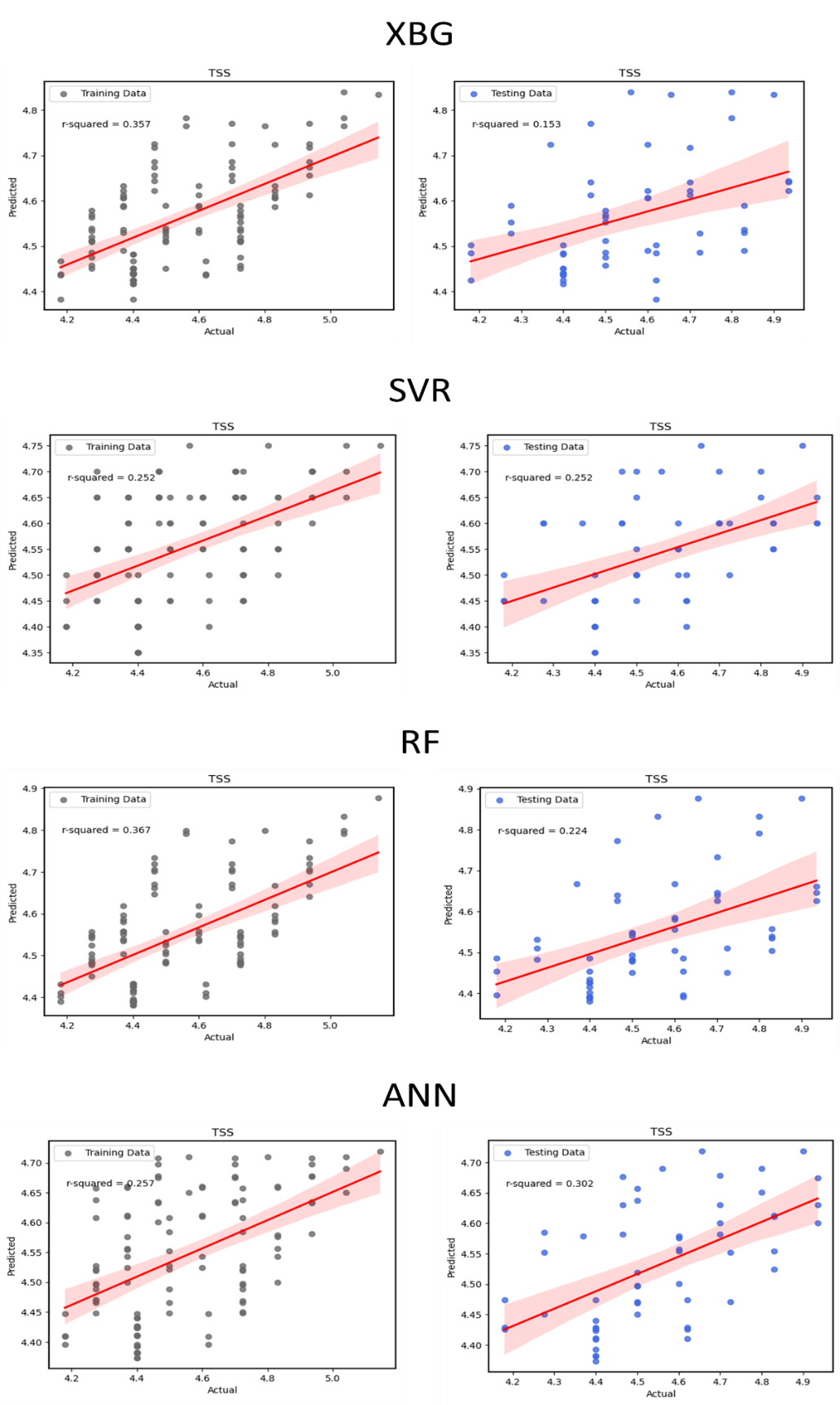 |
| Fig. S2. Comparison between experimental data for garlic's TSS and the predicted values by different ML techniques. |
| 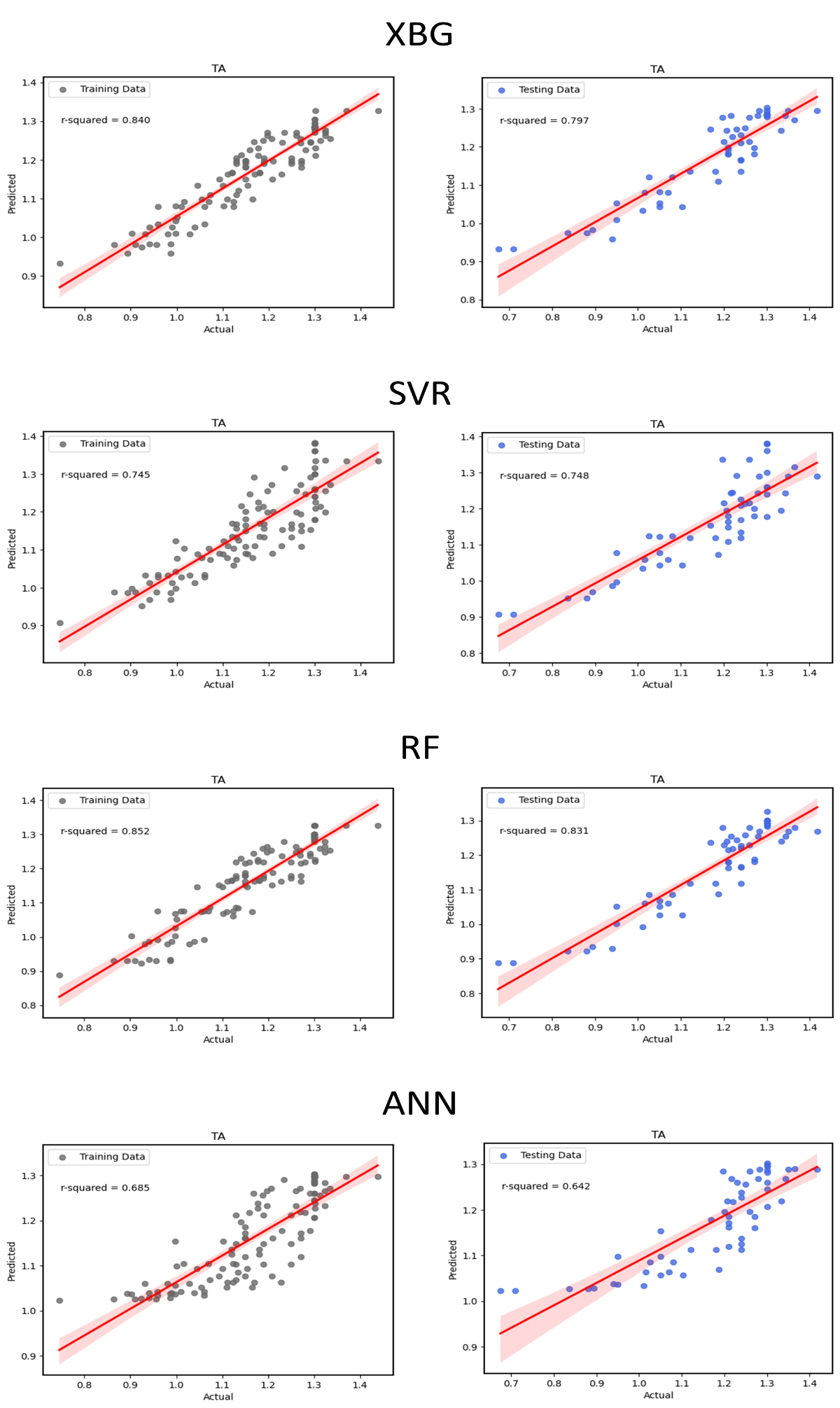 |
| Fig. S3. Comparison between experimental data for garlic's TA and the predicted values by different ML techniques. |
| 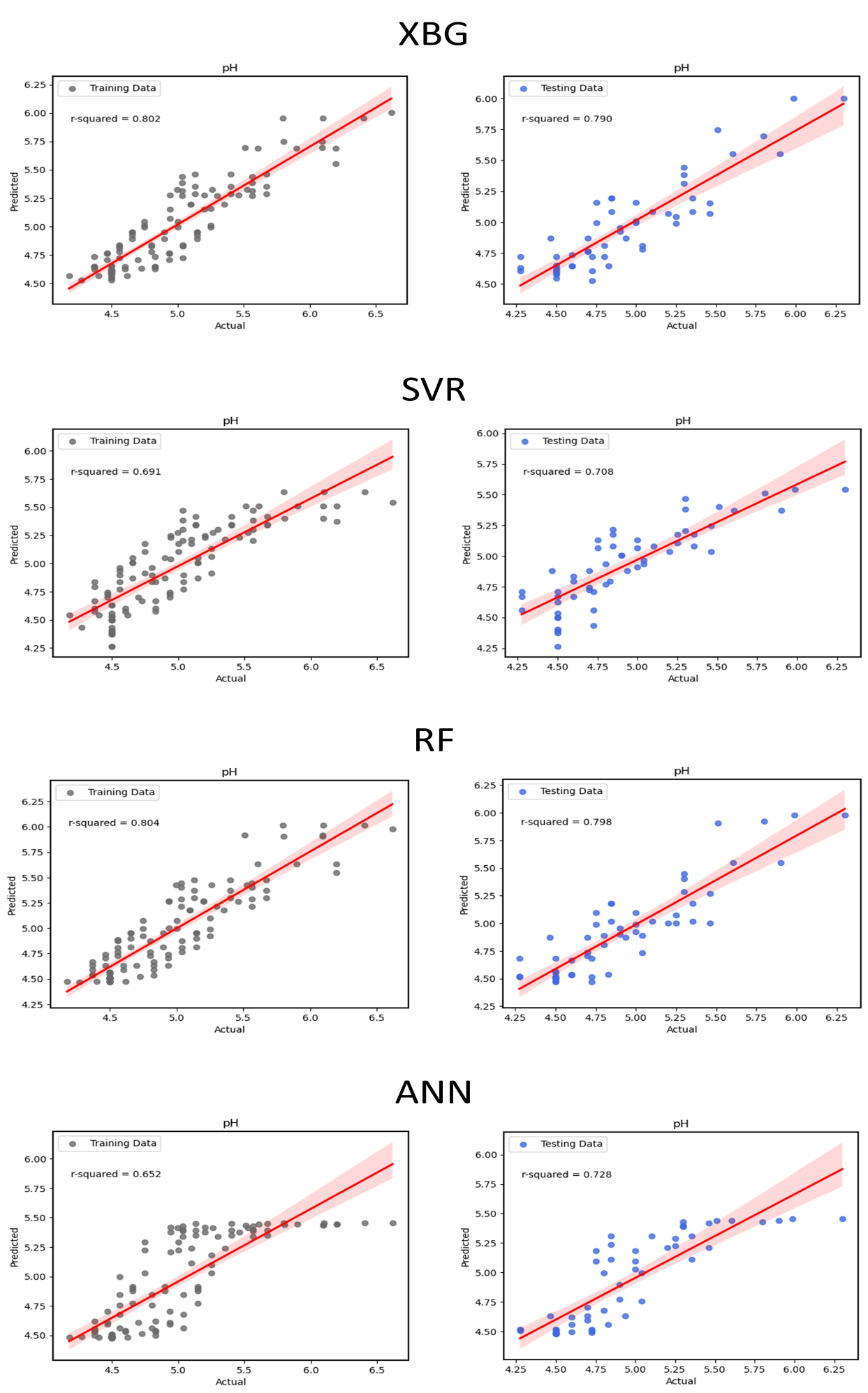 |
| Fig. S4. Comparison between experimental data for garlic's pH and the predicted values by different ML techniques. |

| 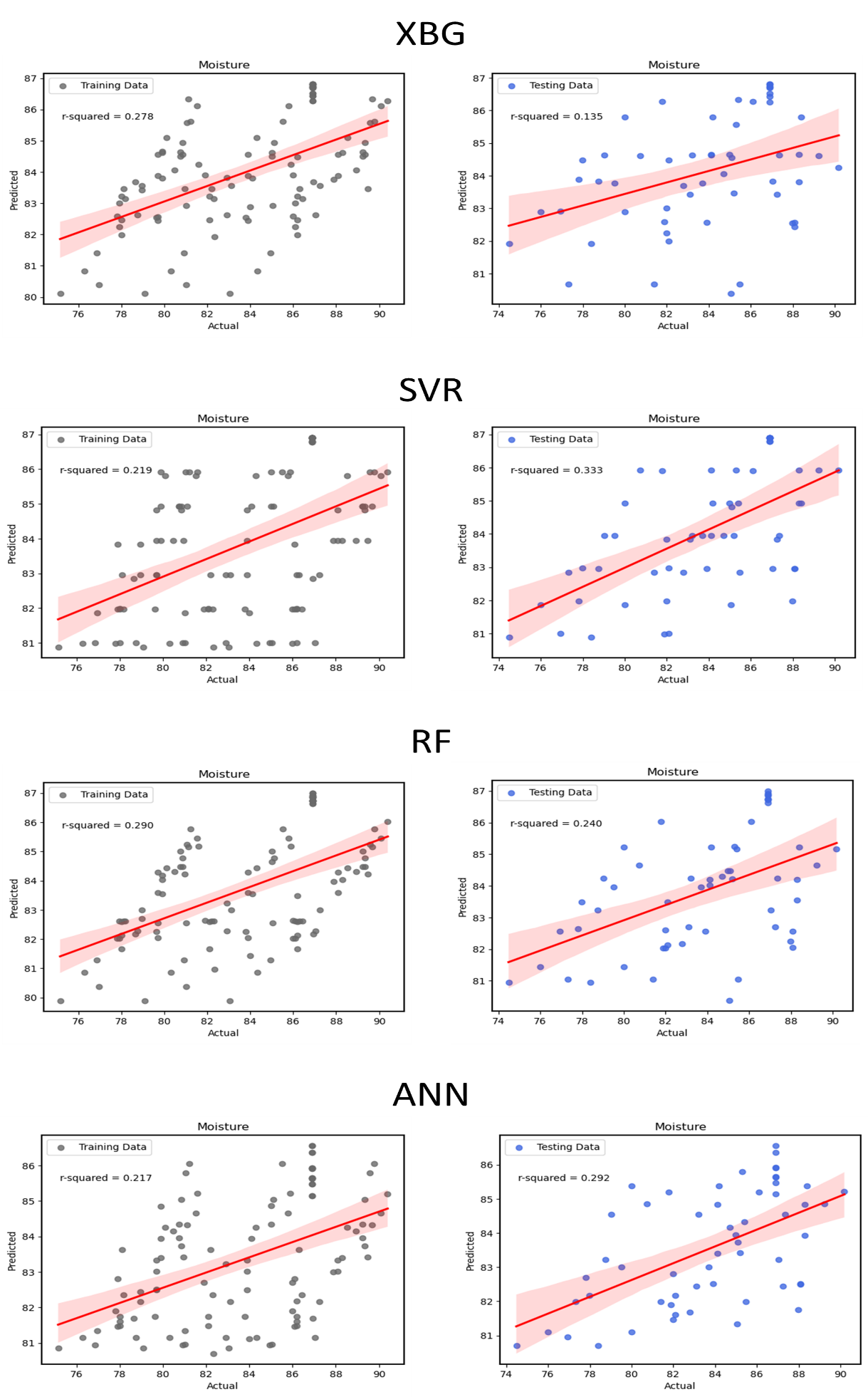 |
| --- |
| Fig. S5. Comparison between experimental data for garlic's moisture content and the predicted values by different ML techniques. |
